# Complete Mitochondrial Genome and Phylogenetic Analysis of *Tarsiger indicus* (Aves: Passeriformes: Muscicapidae)

**DOI:** 10.3390/genes15010090

**Published:** 2024-01-11

**Authors:** Guanwei Lan, Jiaojiao Yu, Juan Liu, Yue Zhang, Rui Ma, Yanshan Zhou, Biqing Zhu, Wei Wei, Jiabin Liu, Guilan Qi

**Affiliations:** 1 Key Laboratory of Southwest China Wildlife Resources Conservation (Ministry of Education), China West Normal University, Nanchong 637009, China; gw.lan@foxmail.com (G.L.); weidamon@163.com (W.W.); 2 Sichuan Key Laboratory of Conservation Biology for Endangered Wildlife, Chengdu Research Base of Giant Panda Breeding, Chengdu 610081, China; yujiaojiao2016@126.com (J.Y.); marui_pandabase@outlook.com (R.M.); zhouyanshan_gsau@163.com (Y.Z.); 3 Administrative Bureau of Baihe National Nature Reserve, Ngawa 623400, China; jzg_lu@163.com (J.L.); zabnsth@163.com (Y.Z.); 18728464191@163.com (B.Z.); 4 Institute of Wildlife Conservation, Central South University of Forestry and Technology, Changsha 410004, China; 5 Animal Husbandry Institute, Chengdu Academy of Agriculture and Forestry Sciences, Chengdu 611130, China

**Keywords:** Muscicapidae, *Tarsiger indicus*, comparative mitogenome, mitogenomic phylogeny

## Abstract

*Tarsiger indicus* (Vieillot, 1817), the White-browed Bush Robin, is a small passerine bird widely distributed in Asian countries. Here, we successfully sequenced its mitogenome using the Illumina Novaseq 6000 platform (Illumina, San Diego, CA, USA) for PE 2 × 150 bp sequencing. Combined with other published mitogenomes, we conducted the first comprehensive comparative mitogenome analysis of Muscicapidae birds and reconstructed the phylogenetic relationships between Muscicapidae and related groups. The *T. indicus* mitogenome was 16,723 bp in size, and it possessed the typical avian mitogenome structure and organization. Most PCGs of *T. indicus* were initiated strictly with the typical start codon ATG, while *COX1* and *ND2* were started with GTG. RSCU statistics showed that CUA, CGA, and GCC were relatively high frequency in the *T. indicus* mitogenome. *T. cyanurus* and *T. indicus* shared very similar mitogenomic features. All 13 PCGs of Muscicapidae mitogenomes had experienced purifying selection. Specifically, *ATP8* had the highest rate of evolution (0.13296), whereas *COX1* had the lowest (0.01373). The monophylies of Muscicapidae, Turdidae, and Paradoxornithidae were strongly supported. The clade of ((Muscicapidae + Turdidae) + Sturnidae) in Passeriformes was supported by both Bayesian Inference and Maximum likelihood analyses. The latest taxonomic status of many passerine birds with complex taxonomic histories were also supported. For example, *Monticola gularis*, *T. indicus*, and *T. cyanurus* were allocated to Turdidae in other literature; our phylogenetic topologies clearly supported their membership in Muscicapidae; *Paradoxornis heudei*, *Suthora webbiana*, *S. nipalensis*, and *S. fulvifrons* were formerly classified into Muscicapidae; we supported their membership in Paradoxornithidae; *Culicicapa ceylonensis* was originally classified as a member of Muscicapidae; our results are consistent with a position in Stenostiridae. Our study enriches the genetic data of *T. indicus* and provides new insights into the molecular phylogeny and evolution of passerine birds.

## 1. Introduction

**Figure 1:**
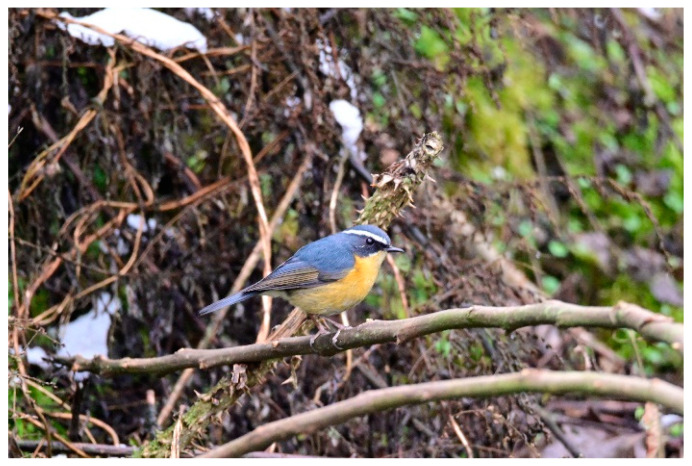
Reference image of adult *T. indicus*. The photo was taken by Taihu Hu on 20 February 2022 in Yingjing County, Ya’an City, Sichuan Province, China.

Passerines (Aves: Passeriformes) include a large number of species and are adapted to various ecological environments. The latest data show that the group has 145 families and 6695 species, accounting for 60% of all bird species; moreover, Muscicapidae is the third-largest family after Tyrannidae and Thraupidae, with 351 species from 53 genera [1]. *Tarsiger indicus* (Vieillot, 1817) (Figure 1), the White-browed Bush Robin, is a small Muscicapidae bird widely distributed in Asian countries, including India, Nepal, Bhutan, Myanmar, Vietnam, and China [2]. In China, *T. indicus* is found in Sichuan, Gansu, Shanxi, Hubei, Yunnan, Tibet, and Taiwan [3,4,5]. It generally inhabits the coniferous forests and the mixed broadleaf–conifer forests between alpine rock valleys at altitudes of 2440–4270 m above sea level in western China; in addition, it also inhabits the bottom shrubland of dense forests at altitudes of 2300–3200 m above sea level in Taiwan Island of China. In the past, the White-browed Bush Robin has been divided into three subspecies, including *T. indicus indicus*, *T. i. yunnanensis*, and *T. i. formosanus* [3]. Recently, an integrative taxonomic investigation found the Taiwan endemic *T. i. formosanus* to be distinctive in genetics, song, and morphology from *T. i. indicus* and *T. i. yunnanensis* of the Sino-Himalayan mountains [6]. In view of this, the *T. i. formosanus* subspecies has been suggested to be upgraded to the species *T. formosanus*, named the Taiwan Bush Robin [6,7]. In addition, *T. indicus* has been included in the updated List of Terrestrial Wild Animals of Important Ecological, Scientific, and Social Value in China [8]. Due to its wide geographical distribution and large population size, the conservation status of *T. indicus* is Least Concern in both the IUCN Red List of Threatened Species [2] and the Red List of China’s Vertebrates [9].

Vertebrate mitochondrial genomes (mitogenomes) are circular, typically 14,000–20,000 bp, and contain 13 protein-coding genes (PCGs), two ribosomal RNA (rRNAs), 22 transfer RNA genes (tRNAs), and one large non-coding D-loop region [10,11]. The mitogenome has been extensively used in population genetics, population dynamics, and adaptive evolution studies of various animal groups [12,13,14,15,16], particularly in phylogenetic reconstruction among animal species [14,16,17,18,19]. It is worth emphasizing that mitochondrial genomes are more reliable in phylogenetic reconstruction than a single mitochondrial gene [20,21,22]. However, the mitogenomes of the Muscicapidae family, a complex lineage of passerines, has been studied very little. So far, complete mitochondrial genomes of only 24 species (ca. 7% of the overall clade) from 15 genera (ca. 28%) within Muscicapidae family have published in the GenBank database (Table 1), mainly focusing on simple mitogenomic descriptions [23,24,25,26,27,28,29].

Genetic data on *T. indicus* are currently rare. In the GenBank database, only 39 nucleotide sequences have been uploaded as of August 2023, including 16 sequences of mitochondrial *Cytb* and *ND2* genes. An accurate understanding of phylogeny is an important prerequisite for many studies of ecology and evolution [6,48]. However, in terms of phylogenetic status, *T. indicus* was previously placed into the genus *Luscinia* [49] and is now still placed into the Turdidae family in some publications [50].

In order to better understand the mitogenome characteristics and the phylogenetic relationship of *T. indicus*, we sequenced its mitochondrial genome through high-throughput sequencing technology here. Combined with other published data, we conduct the first comprehensive comparative mitogenome analysis of Muscicapidae birds and reconstruct the phylogenetic relationships between Muscicapidae and related groups using a mitogenomic approach.

## 2. Materials and Methods

### 2.1. Materials

A subadult window victim, which was found dead, was collected from Yingjing Area of the Giant Panda National Park, Scihuan Province, China (29°33′39.50″ N, 102°51′4.10″ E, 2428 m above sea level) on 30 July 2022, and it was identified as *T. indicus* by morphological characters and mitochondrial *Cytb* blast. The extraction of genomic DNA from a pectoral muscle was carried out using the Rapid Animal Genomic DNA Isolation Kit (Sangon Biotech Co., Ltd., Shanghai, China), according to the manufacturer’s protocol. The specimen and its DNA were deposited at the Chengdu Research Base of Giant Panda Breeding (Dr. Jiabin Liu, jiabin_liu2013@126.com) with the voucher number PB2022027.

### 2.2. Mitogenome Sequencing, Assembly, and Annotation

With the assistance of Sangon Biotech Co., Ltd. (Shanghai, China), we sequenced the mitochondrial genome through a high-throughput sequencing technique. Library preparation, mitogenome sequencing, and mitogenome assembly were performed as previously described [51]. Mitogenome annotations were implemented using MITOS WebServer (http://mitos2.bioinf.uni-leipzig.de/index.py, accessed on 15 August 2023) [52] and MitoAnnotator (http://mitofish.aori.u-tokyo.ac.jp/annotation/input/, accessed on 15 August 2023) [53]. Based on their proposed cloverleaf secondary structures and anticodon sequences, the tRNAs were rechecked using ARWEN online services (http://130.235.244.92/ARWEN/, accessed on 15 August 2023) [54]. The mitogenome visualization map was generated using Chloroplot (https://irscope.shinyapps.io/Chloroplot/, accessed on 18 August 2023) [55].

### 2.3. Comparative Mitogenomic Analyses

The complete mitogenome of *T. indicus* and 24 other Muscicapidae birds belonging to 15 genera were used for comparative mitogenomic analyses (Table 1). The 13 PCGs, two rRNAs, and whole mitogenomes were aligned in batches with MAFFT v7.505 [56]. Nucleotide composition and relative synonymous codon usage (RSCU) were calculated using MEGA v11.0.9 [57]. Nucleotide composition biases were determined from the formulas AT-skew = (A − T)/(A + T) and GC-skew = (G − C)/(G + C). The nucleotide diversity (Pi), the non-synonymous substitution rate (Ka), and the synonymous substitution rate (Ks) were calculated using DnaSP v6.12.03 [58].

Data visualization was performed using OmicStudio tools (https://www.omicstudio.cn/tool, accessed on 25 August 2023) [59].

### 2.4. Mitogenomic Phylogenetic Analyses

Two rRNAs and 13 PCGs of *T. indicus* and 40 other Passeriformes birds belonging to 26 genera and seven families were used for mitogenomic phylogenetic analyses (Table 1). The taxonomy of all birds is based on the IOC World Bird List v13.2 [1]. *Pitta sordida* (Passeriformes: Pittidae) was used as an outgroup based on its well-documented distant phylogenetic position from the ingroup [60,61,62]. Two rRNA sequences were aligned in batches with MAFFT v7.505 [56] using ‘–auto’ strategy and normal alignment mode, and 13 PCGs sequences were aligned in batches using the codon-aware program MACSE v2.06 [63], which preserves reading frame and allows incorporation of sequencing errors or sequences with frameshifts. Ambiguously aligned fragments of these 15 alignments were removed in batches using Gblocks v0.91b [64] with the following parameter settings: minimum number of sequences for a conserved/flank position (22/22), maximum number of contiguous non-conserved positions (8), minimum length of a block (10), allowed gap positions (with half). The 15 alignments were eventually concatenated into one multi-gene dataset consisting of a 13,893 bp sequence using PhyloSuite v1.2.3 [65]. The concatenated multi-gene dataset was used to clarify the phylogeny using Bayesian Inference (BI) and Maximum Likelihood (ML) methods. A best-fit partition model (edge-linked) was selected by ModelFinder v2.2.0 [66] using a BIC criterion, and the results are shown in Table S1. BI phylogenies were inferred using MrBayes v3.2.6 [67] under a partition model (2 parallel runs, ten million generations, sampling every one thousand generations), in which the initial 25% of sampled data were discarded as burn-in. ML phylogenies were inferred using IQ-TREE v2.2.0 [68] under an edge-linked partition model for one hundred thousand ultrafast [69] bootstraps.

High-quality figures of phylogenetic trees were produced using FigTree v.1.4.4 (http://tree.bio.ed.ac.uk/software/figtree/, accessed on 31 August 2023).

## 3. Results and Discussion

### 3.1. Structure and Organization of the T. indicus Mitogenome

**Figure 2:**
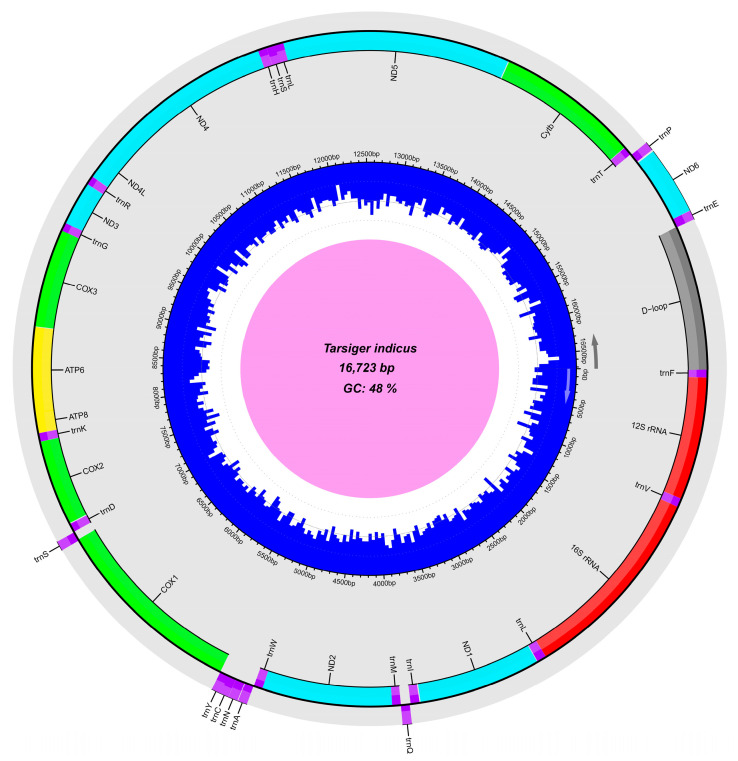
Graphical representation of *Tarsiger indicus* mitogenome. Genes outside the outer multicolored circle are located on the light strand counterclockwise, and those inside the outer circle are located on the heavy strand clockwise. Different colors indicate different types of genes and regions. The inner blue circle represents the local GC content.

Herein, the complete mitogenome of *T. indicus* (GenBank accession number: OR459825) was successfully sequenced and annotated. It was a circular and double-stranded DNA molecule, consisting of a typical structure with 13 PCGs, 2 rRNAs, 22 tRNAs, and a major non-coding D-loop region (Table 2; Figure 2). Among these 37 genes, 28 were located on the heavy strand, while the remaining nine genes, including eight tRNAs (*trnQ*, *trnA*, *trnN*, *trnC*, *trnY*, *trnS2*, *trnE* and *trnP*) and one PCG (*ND6*), were located on the light strand (Table 2; Figure 2). *T. indicus* showed the typical avian mitogenome order [21,70], which was also the ancestral avian arrangement found in many lineages of Passeriformes [21]. The mitogenome structure and organization of *T. indicus* was consistent with those of *T. cyanurus*, but the *T. indicus* mitogenome (16,723 bp) was smaller in size than the *T*. *cyanurus* mitogenome (16,803 bp), and the interspecific difference was mainly caused by the size difference in the D-loop region located between *trnE* and *trnF* (Table 2).

### 3.2. Codon Usage

Among the 13 PCGs, the smallest one was *ATP8*, and the largest one was *ND5*, ranging from 168 bp to 1818 bp (Table 2). Most PCGs of *T. indicus* were initiated with the typical start codon ATG, while *COX1* and *ND2* were started with GTG (Table 2). The unusual start codon GTG was also observed in *COX1* from other bird groups, such as Sittidae [71,72], Accipitridae [73,74], Phasianidae [75], Columbidae [76], and other Passeriformes species [24,25,30,36,45]. The stop codons of 13 PCGs were quite varied in *T. indicus*. *ATP6*, *ATP8*, *COX2*, *Cytb*, *ND3*, *ND4L*, and *ND6* were terminated with the representative stop codon TAA or TAG, *COX1*, *ND1*, and *ND5* ended with AGA or AGG, while *COX3*, *ND2*, and *ND4* were occasionally terminated with the truncated stop codon TA or T (Table 2). The incomplete stop codons TA and T are common in metazoan mitogenomes [19,20,51,72], and they can be converted to TAA by post-transcriptional modifications during the mRNA maturation process [77]. The start and stop codons of the 13 PCGs were very similar in the mitogenomes of *T. indicus* and *T. cyanurus*, and the only difference was the stop codon of the *ND6* gene: the former was TAG, while the latter was AGG (Table 2).

The *T. indicus* mitogenome contained a total of 3797 codons in its protein-coding regions (Table S2). The three most frequently used codons were CUA (Leu1), AUC (Ile), and UUC (Phe), which were used 347, 217, and 181 times, respectively, and the five least-used codons were UGU (Cys), AGU (Ser1), ACG (Thr), CGG (Arg), and AAG (Lys), which were used 6, 6, 6, 4, and 4 times, respectively (Table S2). As in other birds [76,78,79], amino acids with high frequency encoded by PCGs were Leu (664), Thr (327), and Ala (323) (Table S2).

**Figure 3:**
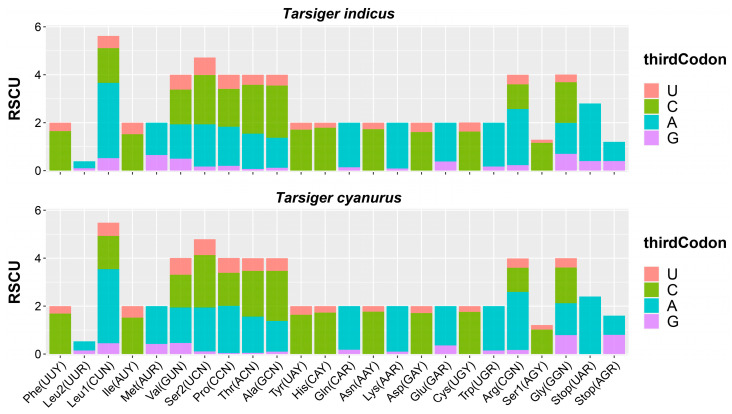
The relative synonymous codon usage (RSCU) in mitogenomes of *T. indicus* and *T. cyanurus*.

In addition, RSCU is a reference value to evaluate the frequency of codons encoding the same amino acid [80]. When the RSCU ratio was greater than 1, it indicated that the codon occurred many times [80]. Statistics on the RSCU showed that CUA (3.14), CGA (2.34), and GCC (2.18) were relatively high-frequency in *T. indicus* mitogenome (Figure 3; Table S2). RSCU values of *T. cyanurus* mitogenome was also summarized and compared with *T. indicus*, and these two mitogenomes had very similar characteristics of utilization rate of synonymous codon of single amino acids (Figure 3; Table S2).

### 3.3. Nucleotide Composition, Diversity, and Evolution

**Figure 4:**
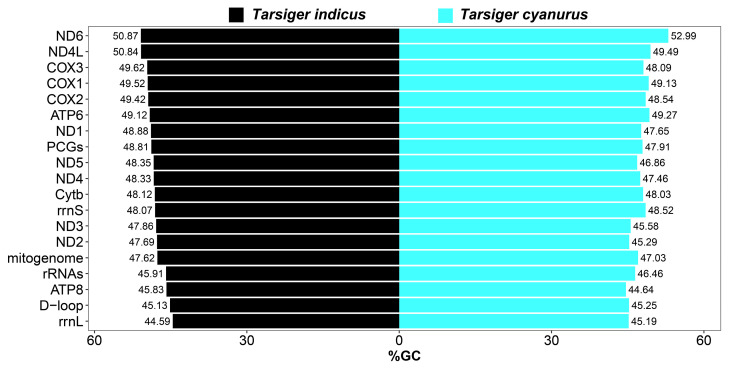
The G + C content (%GC) of *T. indicus* and *T. cyanurus* mitogenomes.

**Figure 5:**
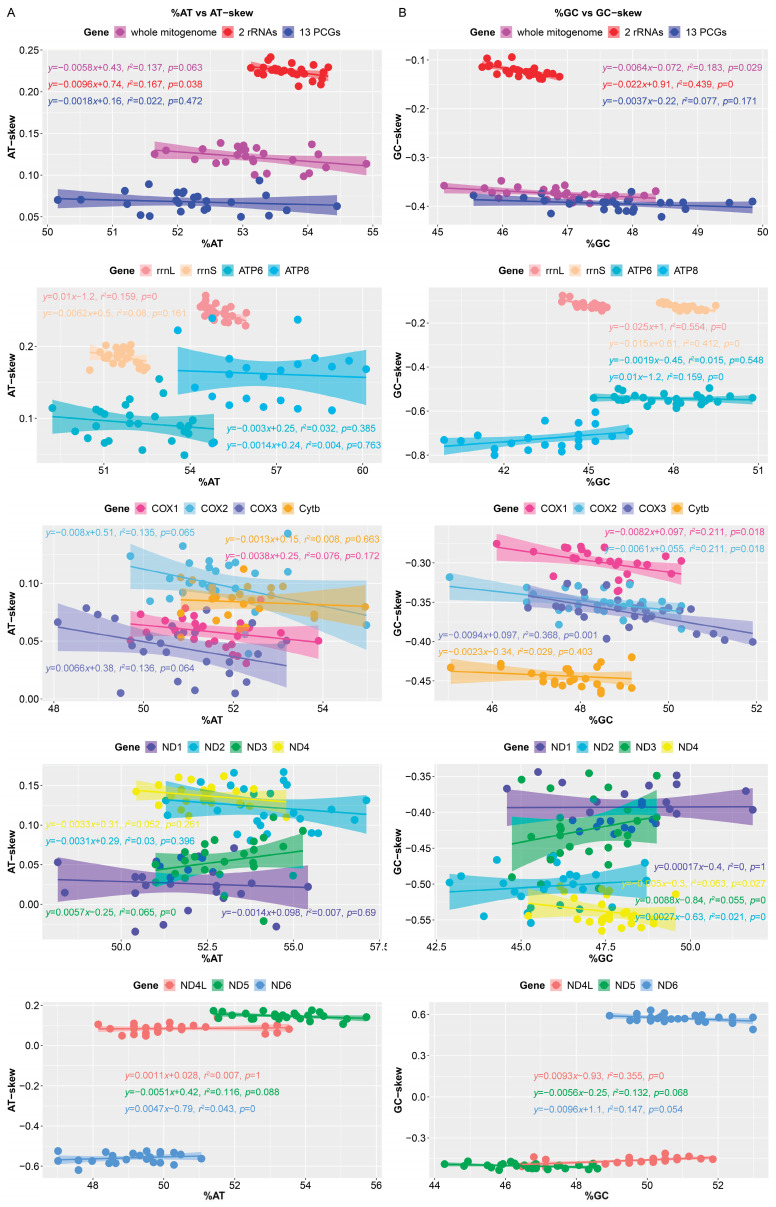
Correlation between nucleotide content and corresponding skew in the mitogenomes of 26 species of Muscicapidae. (**A**) A + T content (%AT) vs. AT-skew; (**B**) G + C content (%GC) vs. GC-skew. Each dot represents a mitogenome.

The overall nucleotide composition of the *T. indicus* mitogenome was 32.88% C, 29.63% A, 22.75% T, and 14.73% G, indicating that the mitogenomes were biased towards C and A bases, which had also been the case in previous studies of avian mitochondrial genomes [18,81]. Its overall G + C content was 47.62%, which was similar to the 47.03% of the *T. cyanurus* mitogenome (Figure 4). Similar to most other birds [18,37,72], overall G + C content of the whole mitogenomes of all 25 Muscicapidae birds was slightly lower than their overall A + T content (Table S3). In terms of a single mitochondrial gene of Muscicapidae species including *T. indicus*, the individual G + C contents were very close to 50% (Table S3; Figure 4 and Figure 5). Although *T. indicus* and *T. cyanurus* were closely related species, their individual G + C content had an inconsistent trend among all genes (Figure 4).

We also calculated the nucleotide skew of mitochondrial gene in 25 Muscicapidae species. The AT-skew values of the entire genome, concatenated rRNAs, concatenated PCGs, and single rRNA and PCG (except *ND6*) were positive, while the GC-skew values were negative (Figure 5), as was common in mitogenomes of Strigiformes [18] and Accipitriformes [74], indicating that Cs were more abundant than Gs, and As were more abundant than Ts. AT-skew and GC-skew were due to the different distribution of nucleotides between the two DNA strands, which further led to an asymmetry in the DNA strands [51,80]. We also analyzed the correlation between nucleotide content and corresponding skew of all mitogenomes of Muscicapidae (Figure 5), but the correlation was weak and further confirmation was needed with more data.

**Figure 6:**
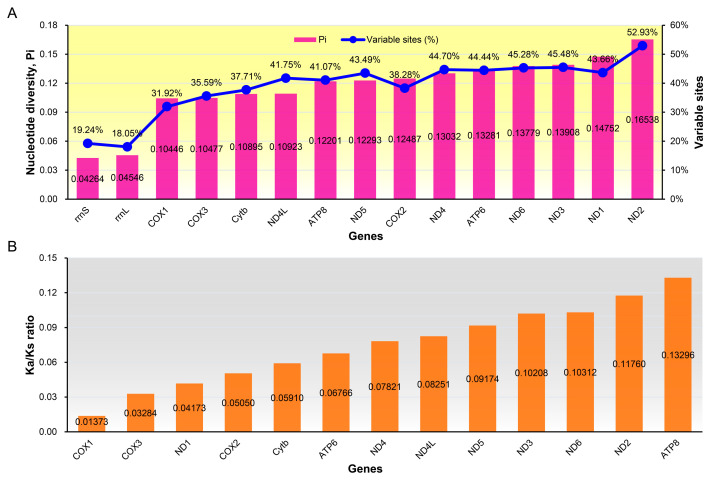
Evolutionary rates of mitochondrial genes of 25 species of Muscicapidae. (**A**) Nucleotide diversity and percentage of variable sites; (**B**)The ratio of non-synonymous substitution rate and synonymous substitution rate.

The nucleotides varied greatly among different genes (Figure 6). The average nucleotide diversity values for individual genes ranged from 0.04264 (*rrnS*) to 0.16538 (*ND2*), and the percentage of nucleotide variable sites ranged from 18.05% (*rrnL*) to 52.93% (*ND2*) (Figure 6A), indicating that *rrnL* and *rrnS* were slow-evolving genes, *ND2* was a fast-evolving gene.

To further understand the role of selective pressure on the mitochondrial PCGs among the Muscicapidae species, we calculated and compared the average Ka/Ks ratio for each PCG (Figure 6B). Ka/Ks ratio = 1 denotes neutral mutations, Ka/Ks ratio < 1 denotes negative selection, and Ka/Ks ratio > 1 denotes positive selection [82,83]. Here, the average Ka/Ks ratio for all PCGs were consistently far lower than 1, indicating that all PCGs of Muscicapidae mitogenomes had experienced purifying selection. Among the 13 PCGs, *ATP8* had the highest rate of evolution (0.13296), whereas *COX1* had the lowest (0.01373) (Figure 6B), which was congruent with the previous studies in Passeriformes [51,71], Piciformes [79], Strigiformes [18], and penguins [84], as well as frogs [85]. Therefore, our findings confirmed that *COX1* experienced the strongest purifying selection and *COX1* might play important roles in the evolution of avian mitogenomes.

### 3.4. Mitochondrial Phylogenomics

**Figure 7:**
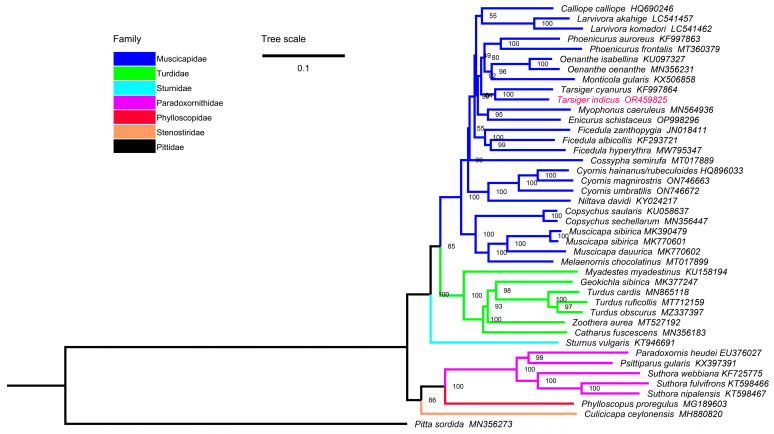
The phylogenetic relationships of Passeriformes inferred by ML method based on the 13PCGs + 2rRNAs dataset. Numbers on nodes are the bootstrap percentages.

The ML and BI trees of the 13PCGs + 2rRNAs dataset had similar topologies, and most nodes were supported by high bootstrap percentages (BP) and Bayesian posterior probabilities (BPP) (Figure 7 and Figure S1).

Our results showed that Muscicapidae, Turdidae, and Paradoxornithidae were clustered into two monophyletic groups, and species of the same genus were clustered together with a high degree of confidence. Muscicapidae and Turdidae were sister groups (BP = 85, BPP = 1.00), and they clustered together with Sturnidae (BP = 100, BPP = 1.00), which was consistent with a previous study [38]. *T. indicus* and *T. cyanurus* were clustered together with high confidence (BP = 100, BPP = 1.00). These two *Tarsiger* birds were previously placed in the genus *Luscinia* [49]. Although many species of Muscicapidae, such as *M. gularis*, *T. indicus*, and *T. cyanurus* were allocated to Turdidae in some older works [50,86] and the up-to-date NCBI taxonomy database; our phylogenetic topologies clearly supported their membership in the Muscicapidae family. It is important to note that the phylogenetic relationships between some genera within Muscicapidae are problematic between our study and a previous study [23]. The position of *C. semirufa* in our ML and BI trees was not consistent, and different from the ML tree based on a 13 PCGs dataset in a Yang et al. study [23], and the degree of confidence of related branches was not high (Figure 7 and Figure S1). Our ML and BI trees showed consistent topology (*Calliope* + *Larvivora*) + *Ficedula* (Figure 7 and Figure S1); however, the ML tree of the Yang et al. study showed the diametrical topology *Calliope* + (*Ficedula* + *Larvivora*) with low bootstrap percentages [23]. Complete mitogenomes may provide more accurate signals than gene fragments for phylogenetic reconstruction. Overall, the current 25 species represent only 7% of the old-world flycatchers group, so, in order to better resolve the phylogenetic relationships within Muscicapidae, it is still necessary to obtain more mitochondrial genome sequences of old-world flycatchers.

In addition, *P. heudei*, *S. webbiana*, *S. nipalensis*, and *S. fulvifrons* were classified into Muscicapidae in previous studies [57,71] and the NCBI taxonomy database, but our results showed that these species clustered into the Paradoxornithidae family [87]. The taxonomic history of *C. ceylonensis* was also complex [72]. *C. ceylonensis* was originally classified into the Muscicapidae family based on external morphology, reproductive habits, and nesting characteristics [86]. Subsequently, it was classified into the family Rhipiduridae [88]. Lately, the phylogenetic analyses based on multilocus sequence data revealed that *C. ceylonensis* was in fact a member of the Stenostiridae family [62]. Here, we also clarified its taxonomic validity based on mitochondrial genome approach.

## 4. Conclusions

In this study, we successfully sequenced the mitogenome of *T. indicus* using the Illumina Novaseq 6000 platform with a paired-end read length of 150 bp. We also annotated and summarized its mitogenomic characteristics in detail. Importantly, we conducted the first comprehensive mitogenome analysis of Muscicapidae. The mitogenome of *T. indicus* mitogenome contained the typical avian mitochondrial gene arrangement. *T. cyanurus* and *T. indicus* shared very similar mitogenomic features. All 13 PCGs of the mitogenomes of Muscicapidae had experienced purifying selection. The monophylies of Muscicapidae, Turdidae, and Paradoxornithidae were strongly supported. The clade of ((Muscicapidae + Turdidae) + Sturnidae) in Passeriformes was supported by both BI and ML analyses. The current taxonomic status of many passerine birds with complex taxonomic histories were also supported. Our study provides the first complete mitochondrial genome of *T. indicus* to enrich its genetic data. A large number of studies on the mitochondrial genome of Muscicapidae are still needed in the future to further solve some phylogenetic problems.

## Figures and Tables

**Table 1:** List of 41 species used for the comparative mitogenomic analyses and the mitogenomic phylogenetic analyses in this study.

Family	Species	GenBank No.		Mitogenome Size (bp)	References
Muscicapidae	*Oenanthe isabellina*	KU097327	NC_040290	16,812	[30]
	*Oenanthe oenanthe*	MN356231	NC_051036	16,826	[31]
	*Copsychus saularis*	KU058637	NC_030603	16,827	[32]
	*Copsychus sechellarum*	MN356447		16,839	[31]
	*Muscicapa sibirica*	MK770601	NC_045374	17,879	[27]
	*Muscicapa sibirica*	MK390479	NC_045181	17,897	[15,28]
	*Muscicapa dauurica*	MK770602	NC_045375	18,026	[29]
	*Ficedula hyperythra*	MW795347	NC_058320	16,819	[23]
	*Ficedula albicollis*	KF293721	NC_021621	16,787	[33]
	*Ficedula zanthopygia*	JN018411	NC_015802	16,794	Unpublished
	*Phoenicurus auroreus*	KF997863	NC_026066	16,772	[34]
	*Phoenicurus frontalis*	MT360379	NC_053917	16,776	[24]
	*Calliope calliope*	HQ690246	NC_015074	16,841	Unpublished
	*Larvivora komadori*	LC541462		16,812	Unpublished
	*Larvivora akahige*	LC541457		16,824	Unpublished
	*Myophonus caeruleus*	MN564936		16,815	Unpublished
	*Enicurus schistaceus*	OP998296	NC_072120	17,112	Unpublished
	*Cyornis umbratilis*	ON746672	NC_068694	16,805	Unpublished
	*Cyornis magnirostris*	ON746663	NC_068687	16,816	Unpublished
	*Cyornis hainanus/rubeculoides*	HQ896033	NC_015232	16,802	[15]
	*Niltava davidi*	KY024217	NC_039538	16,770	[35]
	*Melaenornis chocolatinus*	MT017899	NC_052841	16,582	Unpublished
	*Cossypha semirufa*	MT017889	NC_052839	16,564	Unpublished
	*Tarsiger indicus*	OR459825			This study
	*Tarsiger cyanurus*	KF997864	NC_026067	16,803	[34]
	*Monticola gularis*	KX506858	NC_033536	16,801	[36]
Turdidae	*Turdus ruficollis*	MT712159	NC_057250	16,737	[37]
	*Turdus obscurus*	MZ337397		16,739	[38]
	*Turdus cardis*	MN865118	NC_046948	16,761	[39]
	*Zoothera aurea*	MT527192	NC_054298	16,712	[40]
	*Geokichla sibirica*	MK377247		16,766	[41]
	*Myadestes myadestinus*	KU158194	NC_031352	16,641	[42]
	*Catharus fuscescens*	MN356183	NC_051013	16,766	[31]
Sturnidae	*Sturnus vulgaris*	KT946691	NC_029360	16,793	[43]
Paradoxornithidae	*Suthora fulvifrons*	KT598466	NC_028436	17,059	[44]
	*Suthora nipalensis*	KT598467	NC_028437	16,996	Unpublished
	*Suthora webbiana*	KF725775	NC_024539	16,960	[45]
	*Paradoxornis heudei*	EU376027		16,928	Unpublished
	*Psittiparus gularis*	KX397391	NC_039536	17,109	[35]
Phylloscopidae	*Phylloscopus proregulus*	MG189603	NC_037189	16,880	[46]
Stenostiridae	*Culicicapa ceylonensis*	MH880820	NC_042191	16,851	[47]
Pittidae	*Pitta sordida*	MN356273	NC_051463	17,733	[31]

**Table 2:** The mitochondrial genome comparison between *T. indicus* and *T. cyanurus*.

Gene	Location		Gene Length (bp)		Start/Stop Codon	
	*T. indicus* OR459825	*T. cyanurus* KF997864	*T. indicus* OR459825	*T. cyanurus* KF997864	*T. indicus* OR459825	*T. cyanurus* KF997864
*trnF (gaa)*	1–68: +	1–68: +	68	68		
*rrnS*	69–1050: +	69–1051: +	982	983		
*trnV (uac)*	1051–1120: +	1052–1121: +	70	70		
*rrnL*	1121–2719: +	1122–2723: +	1599	1602		
*trnL2 (uaa)*	2720–2794: +	2724–2798: +	75	75		
*ND1*	2800–3777: +	2804–3781: +	978	978	ATG/AGA	ATG/AGA
*trnI (gau)*	3787–3858: +	3794–3865: +	72	72		
*trnQ (uug)*	3866–3936: −	3873–3943: −	71	71		
*trnM (cau)*	3936–4004: +	3943–4011: +	69	69		
*ND2*	4005–5044: +	4012–5051: +	1040	1040	GTG/TA	GTG/TA
*trnW (uca)*	5045–5115: +	5052–5122: +	71	71		
*trnA (ugc)*	5117–5185: −	5124–5192: −	69	69		
*trnN (guu)*	5190–5262: −	5197–5269: −	73	73		
*trnC (gca)*	5263–5329: −	5270–5336: −	67	67		
*trnY (gua)*	5329–5399: −	5336–5406: −	71	71		
*COX1*	5401–6951: +	5408–6958: +	1551	1551	GTG/AGG	GTG/AGG
*trnS2 (uga)*	6943–7017: −	6950–7024: −	75	75		
*trnD (guc)*	7021–7089: +	7028–7096: +	69	69		
*COX2*	7098–7781: +	7104–7787: +	684	684	ATG/TAA	ATG/TAA
*trnK (uuu)*	7783–7850: +	7789–7856: +	68	68		
*ATP8*	7852–8019: +	7858–8025: +	168	168	ATG/TAA	ATG/TAA
*ATP6*	8010–8693: +	8016–8699: +	684	684	ATG/TAA	ATG/TAA
*COX3*	8699–9482: +	8705–9488: +	784	784	ATG/T	ATG/T
*trnG (ucc)*	9483–9551: +	9489–9557: +	69	69		
*ND3*	9552–9902: +	9558–9908: +	351	351	ATG/TAA	ATG/TAA
*trnR (ucg)*	9904–9973: +	9910–9979: +	70	70		
*ND4L*	9975–10,271: +	9981–10,277: +	297	297	ATG/TAA	ATG/TAA
*ND4*	10,265–11,642: +	10,271–11,648: +	1378	1378	ATG/T	ATG/T
*trnH (gug)*	11,643–11,713: +	11,649–11,719: +	71	71		
*trnS1 (gcu)*	11,714–11,780: +	11,722–11,786: +	67	65		
*trnL1 (uag)*	11,780–11,850: +	11,786–11,856: +	71	71		
*ND5*	11,851–13,668: +	11,857–13,674: +	1818	1818	ATG/AGA	ATG/AGA
*Cytb*	13,677–14,819: +	13,683–14,825: +	1143	1143	ATG/TAA	ATG/TAA
*trnT (ugu)*	14,823–14,891: +	14,829–14,897: +	69	69		
*trnP (ugg)*	14,899–14,968: −	14,904–14,973: −	70	70		
*ND6*	14,982–15,500: −	14,990–15,508: −	519	519	ATG/TAG	ATG/AGG
*trnE (uuc)*	15,502–15,573: −	15,510–15,581: −	72	72		
D-loop	15,574–16,723: +	15,582–16,803: +	1150	1222		

+ represents heavy strand, and − represents light strand.

## Data Availability

The GenBank accession number of the newly determined *Tarsiger indicus* mitogenome sequence is OR459825. The BioProject, BioSample, and SRA accession numbers of metadata are PRJNA1006441, SAMN37041239, and SRR25670941, respectively.

## Supplementary Materials

The following supporting information can be downloaded at: https://www.mdpi.com/article/10.3390/genes15010090/s1, Table S1: The partition and best-fit partition models used in this study; Table S2: The codon usage in the mitogenomes of *T. indicus* and *T. cyanurus*; Table S3: The nucleotide composition and skew in the mitogenomes of 25 species of Muscicapidae; Figure S1: The phylogenetic relationships of Passeriformes inferred by BI method based on the 13PCGs + 2rRNAs dataset. Numbers on nodes are the Bayesian posterior probabilities.
