# Supplementary material for: Complete Mitochondrial Genome and Phylogenetic Analysis of Tarsiger indicus (Aves: Passeriformes: Muscicapidae)
Source: Genes (Basel). 2024 Jan 11;15(1):90. doi: 10.3390/genes15010090 (PMC10815732; doi:10.3390/genes15010090)
Supplement: Supplementary file 1 [file genes-15-00090-s001.zip › genes-2738086-supplementary.pdf]

genes

MDPI

Article

Complete Mitochondrial Genome and Phylogenetic Analysis of *Tarsiger indicus* (Aves: Passeriformes: Muscicapidae)

Guanwei Lan [1,2,†], Jiaojiao Yu [2,†], Juan Liu [3], Yue Zhang [3], Rui Ma [2], Yanshan Zhou [2], Biqing Zhu [3], Wei Wei [1], Jiabin Liu [2,4,*] and Guilan Qi [5,*]

[1] Key Laboratory of Southwest China Wildlife Resources Conservation (Ministry of Education), China West Normal University, Nanchong 637009, China;
[2] Sichuan Key Laboratory of Conservation Biology for Endangered Wildlife, Chengdu Research Base of Giant Panda Breeding, Chengdu 610081, China;
[3] Administrative Bureau of Baihe National Nature Reserve, Ngawa 623400, China;
[4] Institute of Wildlife Conservation, Central South University of Forestry and Technology, Changsha 410004, China
[5] Animal husbandry institute, Chengdu Academy of Agriculture and Forestry Sciences, Chengdu 611130, China
* Correspondence: jiabin_liu2013@126.com (J.L.); qidunwu@163.com (G.Q.)
† These authors contributed equally to this work.

Supplementary Materials

Table S1: The partition and best-fit partition models used in this study;

Table S2: The codon usage in the mitogenomes of *T. indicus* and *T. cyanurus*;

Table S3: The nucleotide composition and skew in the mitogenomes of 25 species of Muscicapidae;

Figure S1: The phylogenetic relationships of Passeriformes inferred by BI method based on the 13PCGs+2rRNAs dataset. Numbers on nodes are the Bayesian posterior probabilities.

Table S1. The partition and best-fit partition models used in this study.

Phylogenetic analysis	Charset	Partition	Model
ML	*ATP6_ATP8_COX2_ND3_ND4*	1-681, 682-846, 2395-3075, 7012-7356, 7357-8733	GTR+F+I+I+R4
	COX1	847-2394	TIM2+F+I+I+R4
	COX3_Cytb_ND4L_ND5	3076-3858, 3859-4998, 8734-9027, 9028-10842	GTR+F+I+I+R4
	ND1	4999-5973	TVM+F+I+I+R4
	ND2	5974-7011	TIM+F+R4
	ND6	10843-11358	TPM2u+F+I+G4
	rrnL_rrnS	11359-12923, 12924-13893	GTR+F+I+I+R3
BI	*ATP6_ATP8_COX2_ND3_ND4*	1-681, 682-846, 2395-3075, 7012-7356, 7357-8733	GTR+F+I+G4
	COX1	847-2394	GTR+F+I+G4
	COX3_Cytb_ND4L_ND5	3076-3858, 3859-4998, 8734-9027, 9028-10842	GTR+F+I+G4
	ND1	4999-5973	GTR+F+I+G4
	ND2	5974-7011	GTR+F+I+G4
	ND6	10843-11358	HKY+F+I+G4
	rrnL_rrnS	11359-12923, 12924-13893	GTR+F+I+G4

Table S2. The codon usage in the mitogenomes of *T. indicus* and *T. cyanurus*.

Code table	Vertebrate Mitochondrial				
Sequences used	2: *Tarsiger indicus* OR459825, *Tarsiger cyanurus* KF997864				
**Codon**	***T. indicus***	***T. cyanurus***	**Codon**	***T. indicus***	***T. cyanurus***
UUU(F)	38 (0.35)	34 (0.31)	UAU(Y)	16 (0.29)	19 (0.36)
UUC(F)	181 (1.65)	187 (1.69)	UAC(Y)	94 (1.71)	88 (1.64)
UUA(L)	32 (0.29)	42 (0.38)	UAA(*)	6 (2.40)	6 (2.40)
UUG(L)	11 (0.10)	16 (0.15)	UAG(*)	1 (0.40)	0 (0.00)
CUU(L)	56 (0.51)	60 (0.55)	CAU(H)	11 (0.21)	14 (0.27)
CUC(L)	160 (1.45)	152 (1.39)	CAC(H)	92 (1.79)	90 (1.73)
CUA(L)	347 (3.14)	339 (3.09)	CAA(Q)	95 (1.86)	92 (1.82)
CUG(L)	58 (0.52)	49 (0.45)	CAG(Q)	7 (0.14)	9 (0.18)
AUU(I)	69 (0.48)	72 (0.48)	AAU(N)	17 (0.27)	15 (0.23)
AUC(I)	217 (1.52)	229 (1.52)	AAC(N)	109 (1.73)	115 (1.77)
AUA(M)	105 (1.35)	126 (1.58)	AAA(K)	83 (1.91)	80 (1.90)
AUG(M)	50 (0.65)	33 (0.42)	AAG(K)	4 (0.09)	4 (0.10)
GUU(V)	30 (0.62)	32 (0.70)	GAU(D)	13 (0.39)	10 (0.29)
GUC(V)	70 (1.45)	63 (1.37)	GAC(D)	53 (1.61)	59 (1.71)
GUA(V)	69 (1.43)	68 (1.48)	GAA(E)	73 (1.62)	73 (1.64)
GUG(V)	24 (0.50)	21 (0.46)	GAG(E)	17 (0.38)	16 (0.36)
UCU(S)	34 (0.73)	31 (0.66)	UGU(C)	6 (0.38)	4 (0.24)
UCC(S)	96 (2.06)	103 (2.19)	UGC(C)	26 (1.63)	30 (1.76)
UCA(S)	82 (1.76)	86 (1.83)	UGA(W)	98 (1.83)	99 (1.85)
UCG(S)	8 (0.17)	5 (0.11)	UGG(W)	9 (0.17)	8 (0.15)
CCU(P)	33 (0.59)	34 (0.62)	CGU(R)	7 (0.40)	7 (0.39)
CCC(P)	88 (1.58)	76 (1.38)	CGC(R)	18 (1.03)	18 (1.01)
CCA(P)	91 (1.63)	109 (1.97)	CGA(R)	41 (2.34)	43 (2.42)
CCG(P)	11 (0.20)	2 (0.04)	CGG(R)	4 (0.23)	3 (0.17)
ACU(T)	34 (0.42)	42 (0.53)	AGU(S)	6 (0.13)	9 (0.19)
ACC(T)	167 (2.04)	151 (1.91)	AGC(S)	54 (1.16)	48 (1.02)
ACA(T)	120 (1.47)	120 (1.51)	AGA(*)	2 (0.80)	2 (0.80)
ACG(T)	6 (0.07)	4 (0.05)	AGG(*)	1 (0.40)	2 (0.80)
GCU(A)	36 (0.45)	43 (0.53)	GGU(G)	18 (0.32)	22 (0.39)
GCC(A)	176 (2.18)	170 (2.09)	GGC(G)	95 (1.70)	83 (1.49)
GCA(A)	101 (1.25)	104 (1.28)	GGA(G)	72 (1.29)	74 (1.33)
GCG(A)	10 (0.12)	8 (0.10)	GGG(G)	39 (0.70)	44 (0.79)
Relative synonymous codon usage is given in parentheses following the codon frequency.					
No. of codons	*Tarsiger indicus*: 3797; *Tarsiger cyanurus*: 3797				

Table S3. The nucleotide composition and skew in the mitogenomes of 25 species of Muscicapidae.

	whole mitogenome									two rRNAs								
Species	T(U)	C	A	G	Size	%GC	GC-skew	%AT	AT-skew	T(U)	C	A	G	Size	%GC	GC-skew	%AT	AT-skew
Calliope calliope HQ690246	23.03	32.87	29.24	14.87	16841	47.73	-0.38	52.27	0.12	20.68	26.34	32.77	20.22	2582	46.55	-0.13	53.45	0.23
Copsychus saularis KU058637	23.31	32.52	29.42	14.74	16827	47.26	-0.38	52.74	0.12	20.55	26.33	32.76	20.36	2579	46.68	-0.13	53.32	0.23
Copsychus sechellarum MN356447	23.29	32.60	29.30	14.81	16836	47.41	-0.38	52.59	0.11	20.70	26.38	32.53	20.39	2585	46.77	-0.13	53.23	0.22
Cossypha semirufa MT017889	24.25	30.98	29.77	15.00	16562	45.98	-0.35	54.02	0.10	21.36	25.24	32.49	20.90	2579	46.14	-0.09	53.86	0.21
Cyornis hainanus/rubeculoides HQ896033	22.91	32.25	30.10	14.74	16802	46.99	-0.37	53.01	0.14	20.77	26.09	32.80	20.34	2576	46.43	-0.12	53.57	0.22
Cyornis magnirostris ON746663	23.08	32.09	30.13	14.69	16816	46.79	-0.37	53.21	0.13	20.58	26.22	33.18	20.03	2571	46.25	-0.13	53.75	0.23
Enicurus schistaceus OP998296	23.33	31.77	30.73	14.17	17112	45.94	-0.38	54.06	0.14	20.93	25.46	33.39	20.23	2561	45.69	-0.11	54.31	0.23
Ficedula albicollis KF293721	22.59	33.10	29.06	15.26	16787	48.35	-0.37	51.65	0.13	20.78	26.29	32.53	20.40	2579	46.68	-0.13	53.32	0.22
Ficedula hyperythra MW795347	22.55	33.19	29.27	14.98	16819	48.18	-0.38	51.82	0.13	20.65	26.24	32.84	20.26	2576	46.51	-0.13	53.49	0.23
Ficedula zanthopygia JN018411	23.48	32.15	29.68	14.68	16794	46.84	-0.37	53.16	0.12	20.84	25.96	32.89	20.30	2581	46.26	-0.12	53.74	0.22
Larvivora akahige akahige LC541457	24.18	31.15	30.10	14.57	16824	45.72	-0.36	54.28	0.11	21.45	25.44	32.75	20.36	2583	45.80	-0.11	54.20	0.21
Larvivora komadori namiyei LC541462	24.33	30.62	30.57	14.48	16812	45.10	-0.36	54.90	0.11	21.30	25.45	32.78	20.48	2578	45.93	-0.11	54.07	0.21
Melaenornis chocolatinus MT017899	23.06	32.32	29.98	14.64	16580	46.96	-0.38	53.04	0.13	20.72	25.96	32.79	20.53	2577	46.49	-0.12	53.51	0.23
Monticola gularis KX506858	23.41	32.33	29.74	14.52	16801	46.85	-0.38	53.15	0.12	21.00	25.83	32.95	20.22	2586	46.06	-0.12	53.94	0.22
Muscicapa sibirica MK390479	23.93	31.89	29.26	14.93	17897	46.82	-0.36	53.18	0.10	20.92	25.89	32.85	20.33	2572	46.23	-0.12	53.77	0.22
Muscicapa latirostris MK770602	24.31	31.63	29.63	14.44	18025	46.06	-0.37	53.94	0.10	20.98	25.81	32.85	20.36	2569	46.17	-0.12	53.83	0.22
Muscicapa sibirica MK770601	23.97	31.84	29.40	14.79	17879	46.63	-0.37	53.37	0.10	20.77	26.06	32.87	20.30	2571	46.36	-0.12	53.64	0.23
Myophonus caeruleus MN564936	23.71	31.45	30.06	14.78	16815	46.23	-0.36	53.77	0.12	21.06	25.09	33.18	20.67	2559	45.76	-0.10	54.24	0.22
Niltava davidi KY024217	23.70	31.44	30.47	14.39	16770	45.84	-0.37	54.16	0.12	20.84	26.00	33.06	20.10	2577	46.10	-0.13	53.90	0.23
Oenanthe isabellina KU097327	22.68	33.00	29.98	14.34	16812	47.34	-0.39	52.66	0.14	20.80	26.27	32.79	20.14	2577	46.41	-0.13	53.59	0.22
Oenanthe oenanthe MN356231	22.91	32.78	30.01	14.29	16825	47.08	-0.39	52.92	0.13	20.49	26.58	32.63	20.29	2577	46.88	-0.13	53.12	0.23
Phoenicurus auroreus KF997863	23.17	32.59	29.75	14.49	16772	47.08	-0.38	52.92	0.12	20.34	26.54	33.05	20.07	2581	46.61	-0.14	53.39	0.24
Phoenicurus frontalis MT360379	23.30	32.36	30.02	14.32	16776	46.68	-0.39	53.32	0.13	20.26	26.50	33.17	20.07	2581	46.57	-0.14	53.43	0.24
Rhinomyias umbratilis ON746672	23.27	31.86	29.77	15.10	16805	46.96	-0.36	53.04	0.12	21.28	25.48	32.94	20.30	2571	45.78	-0.11	54.22	0.22
Tarsiger cyanurus KF997864	23.04	32.67	29.93	14.37	16803	47.03	-0.39	52.97	0.13	20.70	26.23	32.84	20.23	2585	46.46	-0.13	53.54	0.23
Tarsiger indicus OR459825	22.75	32.88	29.63	14.73	16723	47.62	-0.38	52.38	0.13	20.77	25.92	33.32	19.99	2581	45.91	-0.13	54.09	0.23
Avg.	23.37	32.16	29.80	14.66	16928					20.83	25.98	32.89	20.30	2577				

	13 PCGs									rrnL								
Species	T(U)	C	A	G	Size	%GC	GC-skew	%AT	AT-skew	T(U)	C	A	G	Size	%GC	GC-skew	%AT	AT-skew
Calliope calliope HQ690246	23.94	33.95	27.28	14.83	11385	48.77	-0.39	51.23	0.07	20.31	25.81	34.25	19.63	1600	45.44	-0.14	54.56	0.26
Copsychus saularis KU058637	24.47	33.73	27.10	14.70	11388	48.43	-0.39	51.57	0.05	20.65	25.34	34.04	19.96	1598	45.31	-0.12	54.69	0.24
Copsychus sechellarum MN356447	24.38	33.82	27.05	14.75	11388	48.57	-0.39	51.43	0.05	20.65	25.47	33.85	20.03	1598	45.49	-0.12	54.51	0.24
Cossypha semirufa MT017889	25.33	32.01	28.07	14.59	11391	46.60	-0.37	53.40	0.05	21.57	23.70	34.36	20.38	1595	44.08	-0.08	55.92	0.23
Cyornis hainanus/rubeculoides HQ896033	24.00	33.28	28.07	14.64	11391	47.92	-0.39	52.08	0.08	20.53	25.36	34.15	19.96	1593	45.32	-0.12	54.68	0.25
Cyornis magnirostris ON746663	24.21	33.13	28.10	14.56	11400	47.69	-0.39	52.31	0.07	20.32	25.55	34.57	19.56	1585	45.11	-0.13	54.89	0.26
Enicurus schistaceus OP998296	24.14	33.07	29.12	13.67	11394	46.74	-0.41	53.26	0.09	21.08	24.30	34.87	19.75	1580	44.05	-0.10	55.95	0.25
Ficedula albicollis KF293721	23.32	34.65	26.84	15.19	11412	49.84	-0.39	50.16	0.07	20.93	25.19	33.90	19.99	1596	45.18	-0.12	54.82	0.24
Ficedula hyperythra MW795347	23.48	34.46	27.04	15.02	11403	49.49	-0.39	50.51	0.07	20.44	25.58	34.29	19.69	1595	45.27	-0.13	54.73	0.25
Ficedula zanthopygia JN018411	24.68	33.13	27.73	14.46	11391	47.59	-0.39	52.41	0.06	20.66	25.11	34.56	19.66	1597	44.77	-0.12	55.23	0.25
Larvivora akahige akahige LC541457	25.29	32.02	28.39	14.31	11385	46.32	-0.38	53.68	0.06	21.21	24.91	34.11	19.77	1598	44.68	-0.11	55.32	0.23
Larvivora komadori namiyei LC541462	25.52	31.37	28.93	14.18	11385	45.55	-0.38	54.45	0.06	21.15	24.84	34.04	19.96	1598	44.81	-0.11	55.19	0.23
Melaenornis chocolatinus MT017899	24.21	33.32	28.04	14.42	11391	47.75	-0.40	52.25	0.07	20.79	25.11	34.00	20.10	1597	45.21	-0.11	54.79	0.24
Monticola gularis KX506858	24.43	33.66	27.79	14.13	11391	47.78	-0.41	52.22	0.06	20.91	24.97	34.33	19.79	1602	44.76	-0.12	55.24	0.24
Muscicapa sibirica MK390479	24.56	33.21	27.49	14.74	11391	47.95	-0.39	52.05	0.06	20.39	25.61	34.17	19.82	1589	45.44	-0.13	54.56	0.25
Muscicapa latirostris MK770602	25.16	32.81	27.81	14.22	11391	47.03	-0.40	52.97	0.05	20.70	25.24	34.17	19.89	1589	45.12	-0.12	54.88	0.25
Muscicapa sibirica MK770601	24.65	33.09	27.80	14.46	11391	47.55	-0.39	52.45	0.06	20.20	25.74	34.24	19.82	1589	45.56	-0.13	54.44	0.26
Myophonus caeruleus MN564936	24.63	32.67	28.19	14.50	11391	47.18	-0.39	52.82	0.07	21.28	23.94	34.39	20.39	1579	44.33	-0.08	55.67	0.24
Niltava davidi KY024217	24.66	32.38	28.70	14.27	11391	46.64	-0.39	53.36	0.08	20.86	25.00	34.34	19.80	1596	44.80	-0.12	55.20	0.24
Oenanthe isabellina KU097327	23.48	34.43	28.07	14.01	11391	48.44	-0.42	51.56	0.09	20.79	25.30	34.38	19.54	1597	44.83	-0.13	55.17	0.25
Oenanthe oenanthe MN356231	23.94	33.88	28.06	14.12	11399	48.00	-0.41	52.00	0.08	20.25	25.77	34.11	19.87	1595	45.64	-0.13	54.36	0.25
Phoenicurus auroreus KF997863	24.18	33.75	27.80	14.27	11391	48.02	-0.41	51.98	0.07	20.08	25.83	34.46	19.64	1599	45.47	-0.14	54.53	0.26
Phoenicurus frontalis MT360379	24.42	33.45	28.02	14.11	11394	47.56	-0.41	52.44	0.07	19.89	26.02	34.65	19.45	1599	45.47	-0.14	54.53	0.27
Rhinomyias umbratilis ON746672	24.28	32.88	27.74	15.11	11400	47.98	-0.37	52.02	0.07	21.01	24.79	34.45	19.75	1585	44.54	-0.11	55.46	0.24
Tarsiger cyanurus KF997864	23.96	33.77	28.14	14.13	11391	47.91	-0.41	52.09	0.08	20.60	25.41	34.21	19.79	1602	45.19	-0.12	54.81	0.25
Tarsiger indicus OR459825	23.52	34.31	27.67	14.50	11391	48.81	-0.41	51.19	0.08	20.64	25.14	34.77	19.45	1599	44.59	-0.13	55.41	0.26
Avg.	24.34	33.32	27.89	14.46	11393					20.69	25.19	34.29	19.82	1594				

	rrnS									ATP6								
Species	T(U)	C	A	G	Size	%GC	GC-skew	%AT	AT-skew	T(U)	C	A	G	Size	%GC	GC-skew	%AT	AT-skew
Calliope calliope HQ690246	21.28	27.19	30.35	21.18	982	48.37	-0.12	51.63	0.18	22.81	37.72	28.95	10.53	684	48.25	-0.56	51.75	0.12
Copsychus saularis KU058637	20.39	27.93	30.68	21.00	981	48.93	-0.14	51.07	0.20	23.83	37.72	27.19	11.26	684	48.98	-0.54	51.02	0.07
Copsychus sechellarum MN356447	20.77	27.86	30.40	20.97	987	48.83	-0.14	51.17	0.19	23.83	37.28	27.34	11.55	684	48.83	-0.53	51.17	0.07
Cossypha semirufa MT017889	21.04	27.74	29.47	21.75	984	49.49	-0.12	50.51	0.17	25.58	34.80	28.22	11.40	684	46.20	-0.51	53.80	0.05
Cyornis hainanus/rubeculoides HQ896033	21.16	27.26	30.62	20.96	983	48.22	-0.13	51.78	0.18	24.42	35.23	29.68	10.67	684	45.91	-0.54	54.09	0.10
Cyornis magnirostris ON746663	20.99	27.28	30.93	20.79	986	48.07	-0.14	51.93	0.19	24.85	34.50	29.09	11.55	684	46.05	-0.50	53.95	0.08
Enicurus schistaceus OP998296	20.69	27.32	30.99	21.00	981	48.32	-0.13	51.68	0.20	22.37	37.13	30.56	9.94	684	47.08	-0.58	52.92	0.15
Ficedula albicollis KF293721	20.55	28.08	30.32	21.06	983	49.14	-0.14	50.86	0.19	21.79	39.11	27.42	11.69	693	50.79	-0.54	49.21	0.11
Ficedula hyperythra MW795347	21.00	27.32	30.48	21.20	981	48.52	-0.13	51.48	0.18	23.54	37.13	28.36	10.96	684	48.10	-0.54	51.90	0.09
Ficedula zanthopygia JN018411	21.14	27.34	30.18	21.34	984	48.68	-0.12	51.32	0.18	22.95	36.11	30.12	10.82	684	46.93	-0.54	53.07	0.13
Larvivora akahige akahige LC541457	21.83	26.29	30.56	21.32	985	47.61	-0.10	52.39	0.17	23.68	36.70	29.09	10.53	684	47.22	-0.55	52.78	0.10
Larvivora komadori namiyei LC541462	21.53	26.43	30.71	21.33	980	47.76	-0.11	52.24	0.18	25.58	34.80	29.24	10.38	684	45.18	-0.54	54.82	0.07
Melaenornis chocolatinus MT017899	20.61	27.35	30.82	21.22	980	48.57	-0.13	51.43	0.20	23.68	37.13	28.51	10.67	684	47.81	-0.55	52.19	0.09
Monticola gularis KX506858	21.14	27.24	30.69	20.93	984	48.17	-0.13	51.83	0.18	22.81	38.45	28.22	10.53	684	48.98	-0.57	51.02	0.11
Muscicapa sibirica MK390479	21.77	26.35	30.72	21.16	983	47.51	-0.11	52.49	0.17	24.71	36.26	27.63	11.40	684	47.66	-0.52	52.34	0.06
Muscicapa latirostris MK770602	21.43	26.73	30.71	21.12	980	47.86	-0.12	52.14	0.18	24.71	35.82	29.09	10.38	684	46.20	-0.55	53.80	0.08
Muscicapa sibirica MK770601	21.69	26.58	30.65	21.08	982	47.66	-0.12	52.34	0.17	24.85	35.67	28.51	10.96	684	46.64	-0.53	53.36	0.07
Myophonus caeruleus MN564936	20.71	26.94	31.22	21.12	980	48.06	-0.12	51.94	0.20	24.42	35.82	29.24	10.53	684	46.35	-0.55	53.65	0.09
Niltava davidi KY024217	20.80	27.62	30.99	20.59	981	48.22	-0.15	51.78	0.20	24.56	35.67	29.39	10.38	684	46.05	-0.55	53.95	0.09
Oenanthe isabellina KU097327	20.82	27.86	30.20	21.12	980	48.98	-0.14	51.02	0.18	22.66	38.16	29.24	9.94	684	48.10	-0.59	51.90	0.13
Oenanthe oenanthe MN356231	20.88	27.90	30.24	20.98	982	48.88	-0.14	51.12	0.18	23.25	37.72	28.65	10.38	684	48.10	-0.57	51.90	0.10
Phoenicurus auroreus KF997863	20.77	27.70	30.75	20.77	982	48.47	-0.14	51.53	0.19	22.95	38.30	27.05	11.70	684	50.00	-0.53	50.00	0.08
Phoenicurus frontalis MT360379	20.88	27.29	30.75	21.08	982	48.37	-0.13	51.63	0.19	23.39	38.60	27.05	10.96	684	49.56	-0.56	50.44	0.07
Rhinomyias umbratilis ON746672	21.70	26.57	30.53	21.20	986	47.77	-0.11	52.23	0.17	22.51	36.84	28.22	12.43	684	49.27	-0.50	50.73	0.11
Tarsiger cyanurus KF997864	20.85	27.57	30.62	20.96	983	48.52	-0.14	51.48	0.19	23.10	38.01	27.63	11.26	684	49.27	-0.54	50.73	0.09
Tarsiger indicus OR459825	20.98	27.19	30.96	20.88	982	48.07	-0.13	51.93	0.19	22.66	38.16	28.22	10.96	684	49.12	-0.55	50.88	0.11
Avg.	21.05	27.27	30.60	21.08	982					23.67	36.88	28.53	10.91	684				

	ATP8									COX1								
Species	T(U)	C	A	G	Size	%GC	GC-skew	%AT	AT-skew	T(U)	C	A	G	Size	%GC	GC-skew	%AT	AT-skew
Calliope calliope HQ690246	22.62	38.10	32.74	6.55	168	44.64	-0.71	55.36	0.18	23.60	32.62	26.11	17.67	1551	50.29	-0.30	49.71	0.05
Copsychus saularis KU058637	23.81	37.50	30.95	7.74	168	45.24	-0.66	54.76	0.13	24.50	31.40	27.08	17.02	1551	48.42	-0.30	51.58	0.05
Copsychus sechellarum MN356447	24.40	36.90	30.95	7.74	168	44.64	-0.65	55.36	0.12	24.24	31.72	26.89	17.15	1551	48.87	-0.30	51.13	0.05
Cossypha semirufa MT017889	25.60	36.31	29.17	8.93	168	45.24	-0.61	54.76	0.07	24.63	31.01	27.47	16.89	1551	47.90	-0.29	52.10	0.05
Cyornis hainanus/rubeculoides HQ896033	23.81	38.10	32.74	5.36	168	43.45	-0.75	56.55	0.16	23.47	32.24	27.47	16.83	1551	49.07	-0.31	50.93	0.08
Cyornis magnirostris ON746663	23.21	38.69	32.74	5.36	168	44.05	-0.76	55.95	0.17	23.85	32.05	26.86	17.24	1560	49.29	-0.30	50.71	0.06
Enicurus schistaceus OP998296	23.81	38.10	32.74	5.36	168	43.45	-0.75	56.55	0.16	24.05	31.21	28.56	16.18	1551	47.39	-0.32	52.61	0.09
Ficedula albicollis KF293721	25.60	36.90	32.14	5.36	168	42.26	-0.75	57.74	0.11	23.14	32.88	26.79	17.18	1560	50.06	-0.31	49.94	0.07
Ficedula hyperythra MW795347	23.21	39.29	32.14	5.36	168	44.64	-0.76	55.36	0.16	23.59	32.24	26.47	17.69	1560	49.94	-0.29	50.06	0.06
Ficedula zanthopygia JN018411	25.00	36.90	32.14	5.95	168	42.86	-0.72	57.14	0.13	25.34	30.56	26.95	17.15	1551	47.71	-0.28	52.29	0.03
Larvivora akahige akahige LC541457	24.40	35.12	35.12	5.36	168	40.48	-0.74	59.52	0.18	25.02	30.17	28.05	16.76	1551	46.94	-0.29	53.06	0.06
Larvivora komadori namiyei LC541462	25.00	34.52	35.12	5.36	168	39.88	-0.73	60.12	0.17	25.60	29.40	28.30	16.70	1551	46.10	-0.28	53.90	0.05
Melaenornis chocolatinus MT017899	20.83	39.29	33.93	5.95	168	45.24	-0.74	54.76	0.24	24.05	31.59	27.27	17.09	1551	48.68	-0.30	51.32	0.06
Monticola gularis KX506858	24.40	39.29	31.55	4.76	168	44.05	-0.78	55.95	0.13	24.05	31.79	27.14	17.02	1551	48.81	-0.30	51.19	0.06
Muscicapa sibirica MK390479	20.83	39.29	32.74	7.14	168	46.43	-0.69	53.57	0.22	24.50	31.08	26.95	17.47	1551	48.55	-0.28	51.45	0.05
Muscicapa latirostris MK770602	23.81	36.90	33.93	5.36	168	42.26	-0.75	57.74	0.18	24.76	31.01	27.08	17.15	1551	48.16	-0.29	51.84	0.04
Muscicapa sibirica MK770601	20.83	39.29	32.74	7.14	168	46.43	-0.69	53.57	0.22	24.76	30.82	27.21	17.21	1551	48.03	-0.28	51.97	0.05
Myophonus caeruleus MN564936	25.00	35.71	31.55	7.74	168	43.45	-0.64	56.55	0.12	24.56	30.50	27.79	17.15	1551	47.65	-0.28	52.35	0.06
Niltava davidi KY024217	22.02	36.90	35.71	5.36	168	42.26	-0.75	57.74	0.24	24.37	30.95	27.79	16.89	1551	47.84	-0.29	52.16	0.07
Oenanthe isabellina KU097327	23.81	37.50	34.52	4.17	168	41.67	-0.80	58.33	0.18	23.73	32.62	27.40	16.25	1551	48.87	-0.34	51.13	0.07
Oenanthe oenanthe MN356231	26.19	35.12	32.74	5.95	168	41.07	-0.71	58.93	0.11	23.91	32.63	27.24	16.22	1560	48.85	-0.34	51.15	0.07
Phoenicurus auroreus KF997863	24.40	35.12	34.52	5.95	168	41.07	-0.71	58.93	0.17	25.08	31.01	27.01	16.89	1551	47.90	-0.29	52.10	0.04
Phoenicurus frontalis MT360379	23.81	36.90	34.52	4.76	168	41.67	-0.77	58.33	0.18	23.73	32.30	27.40	16.57	1551	48.87	-0.32	51.13	0.07
Rhinomyias umbratilis ON746672	23.81	38.10	33.33	4.76	168	42.86	-0.78	57.14	0.17	25.00	30.71	26.99	17.31	1560	48.01	-0.28	51.99	0.04
Tarsiger cyanurus KF997864	22.62	38.69	32.74	5.95	168	44.64	-0.73	55.36	0.18	23.66	32.56	27.21	16.57	1551	49.13	-0.33	50.87	0.07
Tarsiger indicus OR459825	23.21	39.29	30.95	6.55	168	45.83	-0.71	54.17	0.14	23.53	32.24	26.95	17.28	1551	49.52	-0.30	50.48	0.07
Avg.	23.70	37.45	32.85	6.00	168					24.26	31.51	27.25	16.98	1553				

	COX2									COX3								
Species	T(U)	C	A	G	Size	%GC	GC-skew	%AT	AT-skew	T(U)	C	A	G	Size	%GC	GC-skew	%AT	AT-skew
Calliope calliope HQ690246	21.78	34.80	27.92	15.50	684	50.29	-0.38	49.71	0.12	24.23	33.67	26.28	15.82	784	49.49	-0.36	50.51	0.04
Copsychus saularis KU058637	23.10	33.48	27.49	15.94	684	49.42	-0.36	50.58	0.09	25.38	32.91	25.77	15.94	784	48.85	-0.35	51.15	0.01
Copsychus sechellarum MN356447	22.95	33.63	28.07	15.35	684	48.98	-0.37	51.02	0.10	25.00	33.42	25.77	15.82	784	49.23	-0.36	50.77	0.02
Cossypha semirufa MT017889	25.73	29.68	29.24	15.35	684	45.03	-0.32	54.97	0.06	25.38	32.14	26.53	15.94	784	48.09	-0.34	51.91	0.02
Cyornis hainanus/rubeculoides HQ896033	23.54	31.87	28.51	16.08	684	47.95	-0.33	52.05	0.10	23.98	33.93	26.02	16.07	784	50.00	-0.36	50.00	0.04
Cyornis magnirostris ON746663	22.08	33.19	28.80	15.94	684	49.12	-0.35	50.88	0.13	23.72	34.18	26.02	16.07	784	50.26	-0.36	49.74	0.05
Enicurus schistaceus OP998296	22.81	31.58	30.41	15.20	684	46.78	-0.35	53.22	0.14	23.72	33.93	27.68	14.67	784	48.60	-0.40	51.40	0.08
Ficedula albicollis KF293721	22.95	33.63	27.19	16.23	684	49.85	-0.35	50.15	0.08	22.45	36.35	25.64	15.56	784	51.91	-0.40	48.09	0.07
Ficedula hyperythra MW795347	21.78	34.06	27.92	16.23	684	50.29	-0.35	49.71	0.12	22.70	35.46	26.28	15.56	784	51.02	-0.39	48.98	0.07
Ficedula zanthopygia JN018411	23.39	32.75	28.22	15.64	684	48.39	-0.35	51.61	0.09	25.89	32.65	26.15	15.31	784	47.96	-0.36	52.04	0.00
Larvivora akahige akahige LC541457	23.39	32.31	29.09	15.20	684	47.51	-0.36	52.49	0.11	25.26	31.76	27.93	15.05	784	46.81	-0.36	53.19	0.05
Larvivora komadori namiyei LC541462	23.68	31.14	29.53	15.64	684	46.78	-0.33	53.22	0.11	24.49	32.27	27.93	15.31	784	47.58	-0.36	52.42	0.07
Melaenornis chocolatinus MT017899	24.67	32.41	27.59	15.33	685	47.74	-0.36	52.26	0.06	23.72	33.42	26.91	15.94	784	49.36	-0.35	50.64	0.06
Monticola gularis KX506858	23.10	32.89	28.22	15.79	684	48.68	-0.35	51.32	0.10	23.98	33.93	26.91	15.18	784	49.11	-0.38	50.89	0.06
Muscicapa sibirica MK390479	25.15	31.73	27.19	15.94	684	47.66	-0.33	52.34	0.04	23.72	33.93	26.40	15.94	784	49.87	-0.36	50.13	0.05
Muscicapa latirostris MK770602	24.27	32.75	28.22	14.77	684	47.51	-0.38	52.49	0.08	25.77	31.63	26.53	16.07	784	47.70	-0.33	52.30	0.01
Muscicapa sibirica MK770601	25.29	31.43	27.78	15.50	684	46.93	-0.34	53.07	0.05	23.98	33.67	26.79	15.56	784	49.23	-0.37	50.77	0.06
Myophonus caeruleus MN564936	22.95	32.31	28.95	15.79	684	48.10	-0.34	51.90	0.12	25.26	32.14	27.30	15.31	784	47.45	-0.35	52.55	0.04
Niltava davidi KY024217	23.25	32.75	28.22	15.79	684	48.54	-0.35	51.46	0.10	24.11	33.16	27.04	15.69	784	48.85	-0.36	51.15	0.06
Oenanthe isabellina KU097327	22.51	33.19	28.51	15.79	684	48.98	-0.36	51.02	0.12	22.45	35.84	26.28	15.43	784	51.28	-0.40	48.72	0.08
Oenanthe oenanthe MN356231	23.10	32.60	27.78	16.52	684	49.12	-0.33	50.88	0.09	22.96	35.20	26.40	15.43	784	50.64	-0.39	49.36	0.07
Phoenicurus auroreus KF997863	22.95	32.89	28.65	15.50	684	48.39	-0.36	51.61	0.11	24.62	34.31	24.87	16.20	784	50.51	-0.36	49.49	0.01
Phoenicurus frontalis MT360379	22.95	32.46	29.09	15.50	684	47.95	-0.35	52.05	0.12	24.23	33.93	26.28	15.56	784	49.49	-0.37	50.51	0.04
Rhinomyias umbratilis ON746672	22.81	32.31	28.65	16.23	684	48.54	-0.33	51.46	0.11	24.87	32.27	26.53	16.33	784	48.60	-0.33	51.40	0.03
Tarsiger cyanurus KF997864	22.66	33.04	28.80	15.50	684	48.54	-0.36	51.46	0.12	25.13	32.91	26.79	15.18	784	48.09	-0.37	51.91	0.03
Tarsiger indicus OR459825	22.66	33.33	27.92	16.08	684	49.42	-0.35	50.58	0.10	23.98	33.93	26.40	15.69	784	49.62	-0.37	50.38	0.05
Avg.	23.29	32.62	28.38	15.70	684					24.27	33.58	26.52	15.64	784				

	Cytb									ND1								
Species	T(U)	C	A	G	Size	%GC	GC-skew	%AT	AT-skew	T(U)	C	A	G	Size	%GC	GC-skew	%AT	AT-skew
Calliope calliope HQ690246	24.52	34.24	27.76	13.49	1142	47.72	-0.43	52.28	0.06	25.05	33.64	26.38	14.93	978	48.57	-0.39	51.43	0.03
Copsychus saularis KU058637	22.92	35.61	28.17	13.30	1143	48.91	-0.46	51.09	0.10	26.28	33.23	24.95	15.54	978	48.77	-0.36	51.23	-0.03
Copsychus sechellarum MN356447	22.75	35.87	28.08	13.30	1143	49.17	-0.46	50.83	0.10	26.07	33.44	24.34	16.16	978	49.59	-0.35	50.41	-0.03
Cossypha semirufa MT017889	23.71	34.65	28.43	13.21	1143	47.86	-0.45	52.14	0.09	26.18	31.39	27.61	14.83	978	46.22	-0.36	53.78	0.03
Cyornis hainanus/rubeculoides HQ896033	23.18	34.38	29.05	13.39	1143	47.77	-0.44	52.23	0.11	24.34	34.76	26.07	14.83	978	49.59	-0.40	50.41	0.03
Cyornis magnirostris ON746663	25.02	32.98	28.78	13.21	1143	46.19	-0.43	53.81	0.07	24.85	33.74	26.58	14.83	978	48.57	-0.39	51.43	0.03
Enicurus schistaceus OP998296	23.97	33.77	29.13	13.12	1143	46.89	-0.44	53.11	0.10	24.74	33.33	28.53	13.39	978	46.73	-0.43	53.27	0.07
Ficedula albicollis KF293721	23.53	34.91	27.30	14.26	1143	49.17	-0.42	50.83	0.07	22.80	36.20	25.36	15.64	978	51.84	-0.40	48.16	0.05
Ficedula hyperythra MW795347	24.06	34.91	26.77	14.26	1143	49.17	-0.42	50.83	0.05	23.82	35.38	24.54	16.26	978	51.64	-0.37	48.36	0.01
Ficedula zanthopygia JN018411	23.97	34.65	28.43	12.95	1143	47.59	-0.46	52.41	0.09	24.74	34.15	25.97	15.13	978	49.28	-0.39	50.72	0.02
Larvivora akahige akahige LC541457	24.93	33.07	28.87	13.12	1143	46.19	-0.43	53.81	0.07	28.02	30.57	26.48	14.93	978	45.50	-0.34	54.50	-0.03
Larvivora komadori namiyei LC541462	25.28	32.28	29.66	12.77	1143	45.06	-0.43	54.94	0.08	27.10	30.47	28.32	14.11	978	44.58	-0.37	55.42	0.02
Melaenornis chocolatinus MT017899	23.62	34.21	28.70	13.47	1143	47.68	-0.43	52.32	0.10	24.44	34.46	26.18	14.93	978	49.39	-0.40	50.61	0.03
Monticola gularis KX506858	24.50	34.65	27.65	13.21	1143	47.86	-0.45	52.14	0.06	24.85	34.15	27.10	13.91	978	48.06	-0.42	51.94	0.04
Muscicapa sibirica MK390479	23.53	35.17	28.08	13.21	1143	48.38	-0.45	51.62	0.09	26.18	33.03	25.77	15.03	978	48.06	-0.37	51.94	-0.01
Muscicapa latirostris MK770602	24.23	34.38	28.35	13.04	1143	47.42	-0.45	52.58	0.08	26.38	32.82	27.10	13.70	978	46.52	-0.41	53.48	0.01
Muscicapa sibirica MK770601	24.23	34.47	28.35	12.95	1143	47.42	-0.45	52.58	0.08	26.28	32.92	26.69	14.11	978	47.03	-0.40	52.97	0.01
Myophonus caeruleus MN564936	24.06	34.65	27.56	13.74	1143	48.38	-0.43	51.62	0.07	25.97	32.72	26.79	14.52	978	47.24	-0.39	52.76	0.02
Niltava davidi KY024217	23.88	34.03	28.96	13.12	1143	47.16	-0.44	52.84	0.10	25.26	33.44	27.40	13.91	978	47.34	-0.41	52.66	0.04
Oenanthe isabellina KU097327	23.27	35.52	28.26	12.95	1143	48.47	-0.47	51.53	0.10	24.13	34.66	26.89	14.31	978	48.98	-0.42	51.02	0.05
Oenanthe oenanthe MN356231	23.97	34.65	28.26	13.12	1143	47.77	-0.45	52.23	0.08	24.34	34.36	27.40	13.91	978	48.26	-0.42	51.74	0.06
Phoenicurus auroreus KF997863	23.53	35.43	28.00	13.04	1143	48.47	-0.46	51.53	0.09	25.77	33.54	26.99	13.70	978	47.24	-0.42	52.76	0.02
Phoenicurus frontalis MT360379	24.06	34.21	28.78	12.95	1143	47.16	-0.45	52.84	0.09	24.54	34.15	27.71	13.60	978	47.75	-0.43	52.25	0.06
Rhinomyias umbratilis ON746672	23.36	34.56	28.17	13.91	1143	48.47	-0.43	51.53	0.09	24.85	33.74	25.56	15.85	978	49.59	-0.36	50.41	0.01
Tarsiger cyanurus KF997864	23.71	35.00	28.26	13.04	1143	48.03	-0.46	51.97	0.09	24.64	33.64	27.71	14.01	978	47.65	-0.41	52.35	0.06
Tarsiger indicus OR459825	24.06	34.82	27.82	13.30	1143	48.12	-0.45	51.88	0.07	24.34	34.46	26.79	14.42	978	48.88	-0.41	51.12	0.05
Avg.	23.92	34.50	28.29	13.29	1143					25.23	33.55	26.58	14.63	978				

	ND2									ND3								
Species	T(U)	C	A	G	Size	%GC	GC-skew	%AT	AT-skew	T(U)	C	A	G	Size	%GC	GC-skew	%AT	AT-skew
Calliope calliope HQ690246	23.27	35.10	29.52	12.12	1040	47.21	-0.49	52.79	0.12	25.43	32.57	27.43	14.57	350	47.14	-0.38	52.86	0.04
Copsychus saularis KU058637	24.71	34.52	29.04	11.73	1040	46.25	-0.49	53.75	0.08	24.50	34.47	27.92	13.11	351	47.58	-0.45	52.42	0.07
Copsychus sechellarum MN356447	24.33	34.71	29.04	11.92	1040	46.63	-0.49	53.37	0.09	25.64	33.62	28.21	12.54	351	46.15	-0.46	53.85	0.05
Cossypha semirufa MT017889	24.04	33.56	30.10	12.31	1040	45.87	-0.46	54.13	0.11	26.50	30.48	28.49	14.53	351	45.01	-0.35	54.99	0.04
Cyornis hainanus/rubeculoides HQ896033	22.60	35.29	30.58	11.54	1040	46.83	-0.51	53.17	0.15	25.07	32.76	28.77	13.39	351	46.15	-0.42	53.85	0.07
Cyornis magnirostris ON746663	23.37	34.42	30.96	11.25	1040	45.67	-0.51	54.33	0.14	24.79	33.62	29.34	12.25	351	45.87	-0.47	54.13	0.08
Enicurus schistaceus OP998296	24.69	33.91	31.41	9.99	1041	43.90	-0.54	56.10	0.12	25.36	32.48	29.34	12.82	351	45.30	-0.43	54.70	0.07
Ficedula albicollis KF293721	22.29	36.41	29.01	12.30	1041	48.70	-0.50	51.30	0.13	24.22	33.05	26.78	15.95	351	49.00	-0.35	51.00	0.05
Ficedula hyperythra MW795347	22.79	35.77	28.56	12.88	1040	48.65	-0.47	51.35	0.11	24.79	32.48	29.06	13.68	351	46.15	-0.41	53.85	0.08
Ficedula zanthopygia JN018411	24.71	34.33	29.52	11.44	1040	45.77	-0.50	54.23	0.09	25.50	32.86	28.33	13.31	353	46.18	-0.42	53.82	0.05
Larvivora akahige akahige LC541457	25.36	32.47	30.36	11.82	1041	44.28	-0.47	55.72	0.09	24.79	34.47	26.21	14.53	351	49.00	-0.41	51.00	0.03
Larvivora komadori namiyei LC541462	25.36	32.18	31.41	11.05	1041	43.23	-0.49	56.77	0.11	26.21	31.62	26.78	15.38	351	47.01	-0.35	52.99	0.01
Melaenornis chocolatinus MT017899	23.37	34.90	30.10	11.63	1040	46.54	-0.50	53.46	0.13	25.07	33.62	28.49	12.82	351	46.44	-0.45	53.56	0.06
Monticola gularis KX506858	23.17	35.29	30.67	10.87	1040	46.15	-0.53	53.85	0.14	27.64	31.34	26.50	14.53	351	45.87	-0.37	54.13	-0.02
Muscicapa sibirica MK390479	24.33	33.56	29.71	12.40	1040	45.96	-0.46	54.04	0.10	24.22	34.76	27.64	13.39	351	48.15	-0.44	51.85	0.07
Muscicapa latirostris MK770602	24.62	33.85	30.58	10.96	1040	44.81	-0.51	55.19	0.11	25.64	33.05	28.21	13.11	351	46.15	-0.43	53.85	0.05
Muscicapa sibirica MK770601	24.13	33.85	29.81	12.21	1040	46.06	-0.47	53.94	0.11	24.50	34.47	27.35	13.68	351	48.15	-0.43	51.85	0.05
Myophonus caeruleus MN564936	24.33	34.13	30.67	10.87	1040	45.00	-0.52	55.00	0.12	25.07	32.48	27.35	15.10	351	47.58	-0.37	52.42	0.04
Niltava davidi KY024217	24.81	32.12	32.31	10.77	1040	42.88	-0.50	57.12	0.13	25.07	33.33	30.20	11.40	351	44.73	-0.49	55.27	0.09
Oenanthe isabellina KU097327	22.21	35.87	31.06	10.87	1040	46.73	-0.53	53.27	0.17	24.22	35.61	28.49	11.68	351	47.29	-0.51	52.71	0.08
Oenanthe oenanthe MN356231	23.08	34.71	31.63	10.58	1040	45.29	-0.53	54.71	0.16	24.22	35.04	28.21	12.54	351	47.58	-0.47	52.42	0.08
Phoenicurus auroreus KF997863	23.27	34.81	31.54	10.38	1040	45.19	-0.54	54.81	0.15	25.07	33.62	26.78	14.53	351	48.15	-0.40	51.85	0.03
Phoenicurus frontalis MT360379	25.26	33.33	30.26	11.14	1041	44.48	-0.50	55.52	0.09	25.07	34.19	27.92	12.82	351	47.01	-0.45	52.99	0.05
Rhinomyias umbratilis ON746672	22.88	34.52	30.67	11.92	1040	46.44	-0.49	53.56	0.15	24.79	34.19	26.78	14.25	351	48.43	-0.41	51.57	0.04
Tarsiger cyanurus KF997864	22.79	35.19	31.92	10.10	1040	45.29	-0.55	54.71	0.17	24.22	34.19	30.20	11.40	351	45.58	-0.50	54.42	0.11
Tarsiger indicus OR459825	22.12	36.54	30.19	11.15	1040	47.69	-0.53	52.31	0.15	24.50	34.19	27.64	13.68	351	47.86	-0.43	52.14	0.06
Avg.	23.76	34.44	30.41	11.39	1040					25.08	33.41	28.02	13.50	351				

	ND4									ND4L								
Species	T(U)	C	A	G	Size	%GC	GC-skew	%AT	AT-skew	T(U)	C	A	G	Size	%GC	GC-skew	%AT	AT-skew
Calliope calliope HQ690246	22.79	37.30	28.96	10.96	1378	48.26	-0.55	51.74	0.12	21.89	37.04	27.27	13.80	297	50.84	-0.46	49.16	0.11
Copsychus saularis KU058637	22.21	37.88	29.25	10.67	1378	48.55	-0.56	51.45	0.14	23.23	36.70	25.93	14.14	297	50.84	-0.44	49.16	0.05
Copsychus sechellarum MN356447	22.35	37.88	28.74	11.03	1378	48.91	-0.55	51.09	0.13	22.56	37.04	26.60	13.80	297	50.84	-0.46	49.16	0.08
Cossypha semirufa MT017889	24.38	34.91	30.41	10.30	1378	45.21	-0.54	54.79	0.11	24.92	33.67	28.28	13.13	297	46.80	-0.44	53.20	0.06
Cyornis hainanus/rubeculoides HQ896033	22.42	35.92	29.97	11.68	1378	47.61	-0.51	52.39	0.14	22.56	37.04	27.27	13.13	297	50.17	-0.48	49.83	0.09
Cyornis magnirostris ON746663	22.57	35.99	29.90	11.54	1378	47.53	-0.51	52.47	0.14	22.22	37.37	26.26	14.14	297	51.52	-0.45	48.48	0.08
Enicurus schistaceus OP998296	22.21	36.50	30.77	10.52	1378	47.02	-0.55	52.98	0.16	22.22	36.03	29.29	12.46	297	48.48	-0.49	51.52	0.14
Ficedula albicollis KF293721	21.63	37.52	28.81	12.05	1378	49.56	-0.51	50.44	0.14	23.23	36.70	25.59	14.48	297	51.18	-0.43	48.82	0.05
Ficedula hyperythra MW795347	21.70	38.03	29.39	10.89	1378	48.91	-0.55	51.09	0.15	22.56	37.04	26.94	13.47	297	50.51	-0.47	49.49	0.09
Ficedula zanthopygia JN018411	22.57	37.01	29.32	11.10	1378	48.11	-0.54	51.89	0.13	23.23	36.70	27.27	12.79	297	49.49	-0.48	50.51	0.08
Larvivora akahige akahige LC541457	23.32	35.38	30.12	11.18	1368	46.56	-0.52	53.44	0.13	23.23	36.36	27.95	12.46	297	48.82	-0.49	51.18	0.09
Larvivora komadori namiyei LC541462	23.54	34.94	30.19	11.33	1368	46.27	-0.51	53.73	0.12	23.91	35.69	28.96	11.45	297	47.14	-0.51	52.86	0.10
Melaenornis chocolatinus MT017899	22.50	37.08	30.12	10.30	1378	47.39	-0.57	52.61	0.14	23.57	35.69	25.93	14.81	297	50.51	-0.41	49.49	0.05
Monticola gularis KX506858	22.79	36.72	29.61	10.89	1378	47.61	-0.54	52.39	0.13	24.58	35.02	28.96	11.45	297	46.46	-0.51	53.54	0.08
Muscicapa sibirica MK390479	22.93	36.79	29.68	10.60	1378	47.39	-0.55	52.61	0.13	23.23	36.70	26.26	13.80	297	50.51	-0.45	49.49	0.06
Muscicapa latirostris MK770602	23.08	37.01	28.88	11.03	1378	48.04	-0.54	51.96	0.11	23.23	36.70	26.60	13.47	297	50.17	-0.46	49.83	0.07
Muscicapa sibirica MK770601	22.71	36.87	29.68	10.74	1378	47.61	-0.55	52.39	0.13	22.90	36.70	27.27	13.13	297	49.83	-0.47	50.17	0.09
Myophonus caeruleus MN564936	22.42	36.21	30.19	11.18	1378	47.39	-0.53	52.61	0.15	23.91	35.35	29.29	11.45	297	46.80	-0.51	53.20	0.10
Niltava davidi KY024217	23.00	34.69	30.84	11.47	1378	46.15	-0.50	53.85	0.15	24.92	34.34	27.95	12.79	297	47.14	-0.46	52.86	0.06
Oenanthe isabellina KU097327	22.13	37.52	29.61	10.74	1378	48.26	-0.55	51.74	0.14	21.55	37.71	26.60	14.14	297	51.85	-0.45	48.15	0.10
Oenanthe oenanthe MN356231	21.92	37.45	30.12	10.52	1378	47.97	-0.56	52.03	0.16	22.90	36.03	27.61	13.47	297	49.49	-0.46	50.51	0.09
Phoenicurus auroreus KF997863	22.42	36.72	30.19	10.67	1378	47.39	-0.55	52.61	0.15	22.90	36.36	27.95	12.79	297	49.16	-0.48	50.84	0.10
Phoenicurus frontalis MT360379	22.86	36.14	30.48	10.52	1378	46.66	-0.55	53.34	0.14	22.90	36.36	26.94	13.80	297	50.17	-0.45	49.83	0.08
Rhinomyias umbratilis ON746672	22.93	35.70	29.68	11.68	1378	47.39	-0.51	52.61	0.13	22.22	36.70	27.95	13.13	297	49.83	-0.47	50.17	0.11
Tarsiger cyanurus KF997864	22.79	36.72	29.75	10.74	1378	47.46	-0.55	52.54	0.13	23.23	35.69	27.27	13.80	297	49.49	-0.44	50.51	0.08
Tarsiger indicus OR459825	21.70	37.30	29.97	11.03	1378	48.33	-0.54	51.67	0.16	22.22	36.70	26.94	14.14	297	50.84	-0.44	49.16	0.10
Avg.	22.61	36.62	29.79	10.98	1377					23.08	36.29	27.35	13.29	297				

	ND5									ND6								
Species	T(U)	C	A	G	Size	%GC	GC-skew	%AT	AT-skew	T(U)	C	A	G	Size	%GC	GC-skew	%AT	AT-skew
Calliope calliope HQ690246	22.06	36.03	29.87	12.05	1818	48.07	-0.50	51.93	0.15	37.76	10.98	10.21	41.04	519	52.02	0.58	47.98	-0.57
Copsychus saularis KU058637	22.77	35.70	29.76	11.77	1818	47.47	-0.50	52.53	0.13	38.57	10.47	10.08	40.89	516	51.36	0.59	48.64	-0.59
Copsychus sechellarum MN356447	22.77	35.64	29.70	11.88	1818	47.52	-0.50	52.48	0.13	38.57	10.47	11.05	39.92	516	50.39	0.58	49.61	-0.55
Cossypha semirufa MT017889	24.59	33.06	30.47	11.88	1818	44.94	-0.47	55.06	0.11	35.84	13.49	11.18	39.50	519	52.99	0.49	47.01	-0.52
Cyornis hainanus/rubeculoides HQ896033	22.88	34.38	30.86	11.88	1818	46.26	-0.49	53.74	0.15	38.34	10.98	10.60	40.08	519	51.06	0.57	48.94	-0.57
Cyornis magnirostris ON746663	22.55	34.82	31.13	11.50	1818	46.31	-0.50	53.69	0.16	38.34	11.18	11.56	38.92	519	50.10	0.55	49.90	-0.54
Enicurus schistaceus OP998296	22.33	35.15	31.85	10.67	1818	45.82	-0.53	54.18	0.18	39.31	10.79	10.40	39.50	519	50.29	0.57	49.71	-0.58
Ficedula albicollis KF293721	22.28	36.08	29.26	12.38	1818	48.46	-0.49	51.54	0.14	37.38	12.14	10.21	40.27	519	52.41	0.54	47.59	-0.57
Ficedula hyperythra MW795347	21.25	37.01	30.15	11.59	1821	48.60	-0.52	51.40	0.17	38.54	10.98	9.06	41.43	519	52.41	0.58	47.59	-0.62
Ficedula zanthopygia JN018411	22.99	34.87	30.47	11.66	1818	46.53	-0.50	53.47	0.14	38.34	10.60	11.56	39.50	519	50.10	0.58	49.90	-0.54
Larvivora akahige akahige LC541457	23.93	33.72	31.24	11.11	1818	44.83	-0.50	55.17	0.13	38.15	10.79	11.75	39.31	519	50.10	0.57	49.90	-0.53
Larvivora komadori namiyei LC541462	23.93	33.00	31.79	11.28	1818	44.28	-0.49	55.72	0.14	38.54	10.98	11.75	38.73	519	49.71	0.56	50.29	-0.53
Melaenornis chocolatinus MT017899	22.66	34.54	31.74	11.06	1818	45.60	-0.52	54.40	0.17	39.50	9.25	10.40	40.85	519	50.10	0.63	49.90	-0.58
Monticola gularis KX506858	23.10	35.59	30.14	11.17	1818	46.75	-0.52	53.25	0.13	38.92	10.98	11.56	38.54	519	49.52	0.56	50.48	-0.54
Muscicapa sibirica MK390479	22.72	34.93	30.42	11.94	1818	46.86	-0.49	53.14	0.14	38.15	10.98	11.18	39.69	519	50.67	0.57	49.33	-0.55
Muscicapa latirostris MK770602	23.82	34.32	30.31	11.55	1818	45.87	-0.50	54.13	0.12	39.88	10.02	11.18	38.92	519	48.94	0.59	51.06	-0.56
Muscicapa sibirica MK770601	22.72	35.04	30.47	11.77	1818	46.81	-0.50	53.19	0.15	37.76	11.37	11.75	39.11	519	50.48	0.55	49.52	-0.53
Myophonus caeruleus MN564936	22.99	34.21	31.08	11.72	1818	45.93	-0.49	54.07	0.15	37.76	11.56	10.79	39.88	519	51.45	0.55	48.55	-0.56
Niltava davidi KY024217	23.27	34.10	31.24	11.39	1818	45.49	-0.50	54.51	0.15	38.73	9.83	10.79	40.66	519	50.48	0.61	49.52	-0.56
Oenanthe isabellina KU097327	21.84	36.08	30.97	11.11	1818	47.19	-0.53	52.81	0.17	38.73	10.79	10.79	39.69	519	50.48	0.57	49.52	-0.56
Oenanthe oenanthe MN356231	22.66	35.42	30.69	11.22	1818	46.64	-0.52	53.36	0.15	38.42	10.42	10.04	41.12	518	51.54	0.60	48.46	-0.59
Phoenicurus auroreus KF997863	22.00	36.30	30.31	11.39	1818	47.69	-0.52	52.31	0.16	36.99	11.75	11.37	39.88	519	51.64	0.54	48.36	-0.53
Phoenicurus frontalis MT360379	22.66	35.59	30.47	11.28	1818	46.86	-0.52	53.14	0.15	40.08	9.83	10.21	39.88	519	49.71	0.60	50.29	-0.59
Rhinomyias umbratilis ON746672	23.32	33.99	30.53	12.16	1818	46.15	-0.47	53.85	0.13	37.57	10.79	10.98	40.66	519	51.45	0.58	48.55	-0.55
Tarsiger cyanurus KF997864	22.44	35.64	30.69	11.22	1818	46.86	-0.52	53.14	0.16	36.99	11.18	10.02	41.81	519	52.99	0.58	47.01	-0.57
Tarsiger indicus OR459825	21.45	36.96	30.20	11.39	1818	48.35	-0.53	51.65	0.17	37.76	10.98	11.37	39.88	519	50.87	0.57	49.13	-0.54
Avg.	22.77	35.08	30.61	11.54	1818					38.27	10.91	10.84	39.99	519				

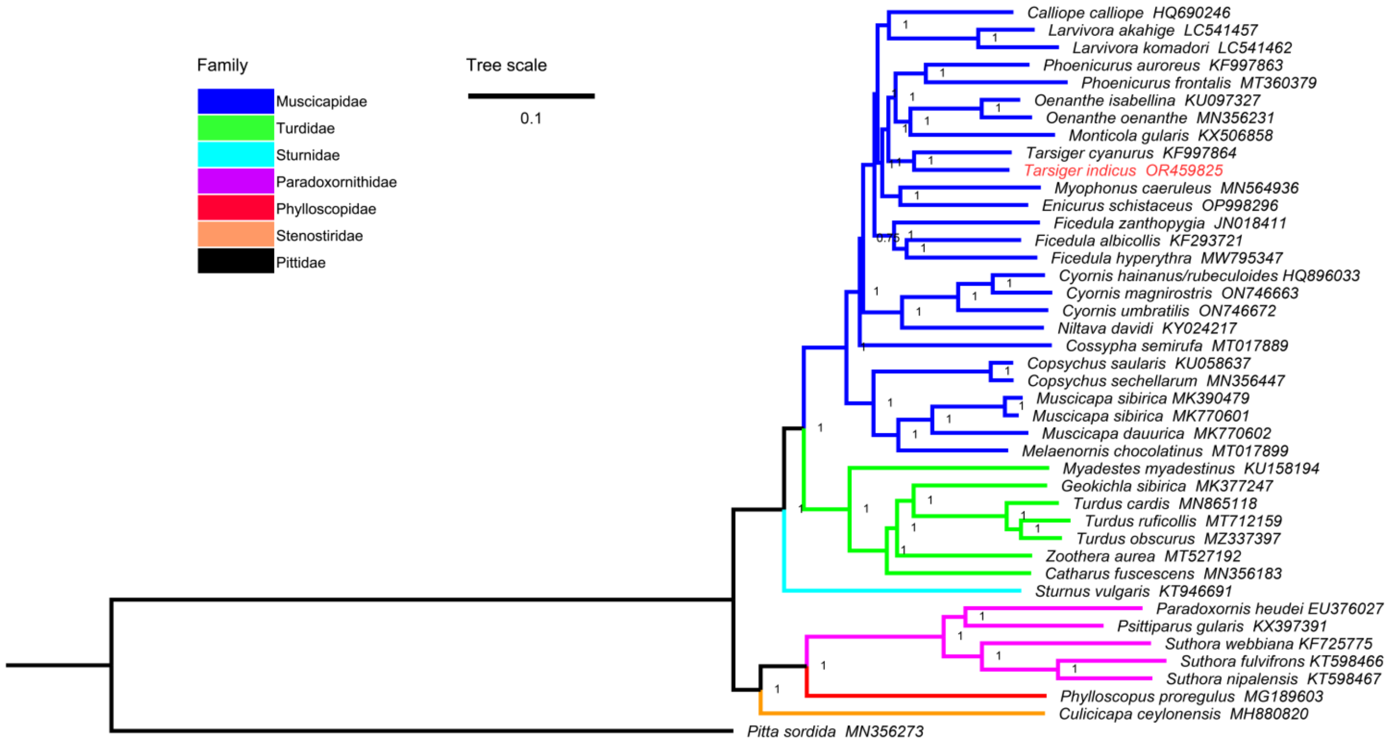

Figure S1. The phylogenetic relationships of Passeriformes inferred by BI method based on the 13PCGs+2rRNAs dataset. Numbers on nodes are the Bayesian posterior probabilities.
